# Neo-intline: integrated pipeline enables neoantigen design through the *in-silico* presentation of T-cell epitope

**DOI:** 10.1038/s41392-023-01644-9

**Published:** 2023-10-18

**Authors:** Bingyu Li, Ping Jing, Genhui Zheng, Chenyu Pi, Lu Zhang, Zuojing Yin, Lijun Xu, Jingxuan Qiu, Hua Gu, Tianyi Qiu, Jianmin Fang

**Affiliations:** 1 Laboratory of Molecular Medicine, Shanghai Key Laboratory of Signaling and Disease Research, School of Life Sciences and Technology, Tongji Hospital, Tongji University Suzhou Institute, Tongji University, Shanghai, China; 2 School of Basic Medical Sciences, Henan University of Science and Technology, Luoyang, Henan China; 3 Institute of Clinical Science, Zhongshan Hospital, Fudan University, Shanghai, China; 4 Oden Institute for Computational Engineering and Sciences (ICES), University of Texas at Austin, Austin, TX USA; 5 School of Health Science and Engineering, University of Shanghai for Science and Technology, Shanghai, China; 6 Shanghai Institute of Infectious Disease and Biosecurity, Fudan University, Shanghai, 200032 China

**Keywords:** Immunotherapy, Skin cancer, Genome informatics

## Abstract

Neoantigen vaccines are one of the most effective immunotherapies for personalized tumour treatment. The current immunogen design of neoantigen vaccines is usually based on whole-genome sequencing (WGS) and bioinformatics prediction that focuses on the prediction of binding affinity between peptide and MHC molecules, ignoring other peptide-presenting related steps. This may result in a gap between high prediction accuracy and relatively low clinical effectiveness. In this study, we designed an integrated *in-silico* pipeline, Neo-intline, which started from the SNPs and indels of the tumour samples to simulate the presentation process of peptides in-vivo through an integrated calculation model. Validation on the benchmark dataset of TESLA and clinically validated neoantigens illustrated that neo-intline could outperform current state-of-the-art tools on both sample level and melanoma level. Furthermore, by taking the mouse melanoma model as an example, we verified the effectiveness of 20 neoantigens, including 10 MHC-I and 10 MHC-II peptides. The in-vitro and in-vivo experiments showed that both peptides predicted by Neo-intline could recruit corresponding CD4^+^ T cells and CD8^+^ T cells to induce a T-cell-mediated cellular immune response. Moreover, although the therapeutic effect of neoantigen vaccines alone is not sufficient, combinations with other specific therapies, such as broad-spectrum immune-enhanced adjuvants of granulocyte-macrophage colony-stimulating factor (GM-CSF) and polyinosinic-polycytidylic acid (poly(I:C)), or immune checkpoint inhibitors, such as PD-1/PD-L1 antibodies, can illustrate significant anticancer effects on melanoma. Neo-intline can be used as a benchmark process for the design and screening of immunogenic targets for neoantigen vaccines.

## Introduction

Cancer is an increasing global burden, with over 10 million new cases and millions of deaths annually,^1^ and its incidence will continue to increase to ~28.4 million new cases by 2040 according to model estimation.^2^ As a new cancer therapy, immunotherapy has been demonstrated to be an advanced strategy for eliminating cancer cells by triggering the immune system of patients.^3,4^ Therapeutic vaccines, a type of immunotherapy strategy,^3,4^ which have been proven to be significantly effective for cancers such as pancreatic ductal adenocarcinoma^5^ and melanoma,^6,7^ requires tumour-specific neopeptides or so-called neoantigens. Neoantigens are recognized and bonded by the major histocompatibility complexes (MHC) to form the MHC-neoantigen complexes, which are further recognized by the T cells and trigger the personalized immune response of the individual patient.^8,9^ Personalized neoantigen therapy could induce long-lasting tumour-specific memory T cells through de novo induction of the T-cell population, boosting the existing T-cell response and epitope spreading.^9^

The screening of cancer-specific neoantigens is dependent on multi-omics data and bioinformatics prediction of T-cell epitope.^3^ Currently, tumour-specific neoantigens are mainly designed through standard *in-silico* pipelines, which follow two rules. First, the neoantigen peptides should contain the tumour-specific mutation, which means that the personalized somatic mutations, which occur on the cancer cell rather than the normal cell, were filtered to generate the target neoantigen peptide. Second, mutation-containing peptides (MCPs) should be presented by the MHC and activate the T-cell-mediated immune response.^10^ Although the current benchmark method of T-cell epitope prediction can reach a high accuracy of 80%-90%,^11^ only several neoantigen vaccines, like NeoVax^12^ and Neo-MoDC,^13^ have been approved by the Food and Drug Administration (FDA) or in clinical trials. The gap between high prediction accuracy and low clinical effects is likely due to the complex immune presentation processes.

Current standard *in-silico* pipelines focus on the binding affinity prediction between mutation-containing peptide (MCP) and major histocompatibility complex (MHC), ignoring the other steps of the presenting processes, including proteasome hydrolysis,^14^ the transport efficiency of transporters associated with antigen processing,^15^ and T-cell receptor (TCR) recognition.^16^ Additionally, for MCP and MHC binding, the above pipelines only considered the prediction of endogenous peptide presentation, while more recent research pointed out that in addition to MHC-I peptides, CD4^+^ T-cell neoepitopes (MHC class II epitopes) could also reshape the tumour microenvironment and drive the therapeutic T-cell immune response to cancer.^17^ Considering it is necessary to ensure that the neoantigen can successfully go past all the presenting steps, the pipelines of neoantigen prediction should contain the comprehensive steps of presenting processes to generate suitable MHC-I and MHC-II neoantigen targets for preclinical experiments.

To achieve this purpose, we presented a novel computational pipeline, Neo-intline (integrated pipline of neoantigen design), to generate personalized neoantigens from the next generation sequencing (NGS) data. This pipeline integrates the processes of T-cell immune presentations, which considers single nucleotide polymorphisms (SNPs) and insertion and deletion (Indel) data from NGS. The prediction ability of Neo-intline was systemically evaluated on seven cancer patients from clinical trials,^18–20^ TESLA community data^21^ and literature datasets.^22,23^ Neo-intline was compared with other state-of-the-art peers, which illustrated good performance on melanoma. In addition, we tested the effectiveness of Neo-intline-generated neoantigens in mouse melanoma models. The top 10 MHC-I peptides and top 10 MHC-II peptides from the ranking list were synthesized for both in-vitro and in-vivo experimental validation. The results showed that the neoantigens of melanoma generated through Neo-intline could (1) stimulate the activation of T cells in-vitro, (2) inhibit tumour growth in mice by combining with adjuvants such as GM-CSF and Poly (I:C), and (3) illustrate anticancer ability through combination therapy with immune checkpoint inhibitors of anti-PD1/anti-PDL1 antibodies.

## Results

### Model performance of Neo-intline compared with state-of-the-art peers

**Fig. 1:**
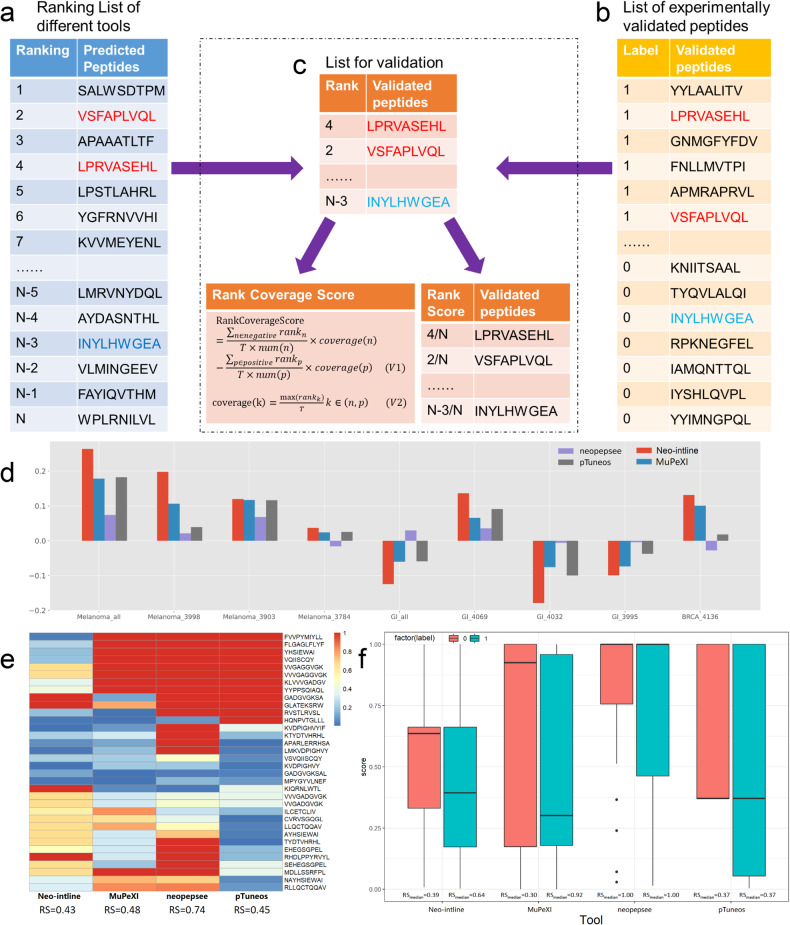
The model evaluation based on Rank Coverage Score (RCS) and Rank Score (RS). **a** Ranking List of different tools including Neo-intline, MuPeXi, Neopepsee, and pTuneos. **b** Experimentally validated peptides derived from three benchmarks^18–20^ and IEDB.^27^ **c** List for validation and two evaluation parameters. **d** Rank coverage score (RCS) of the final rank list obtained from Neo-intline, MuPeXI, neopepsee and pTuneos on 7 clinical samples, overall melanoma samples, and overall GI samples. **e** Ranking score (RS) on all positive positive neoantigens calculated by four compared algorithms. **f** Boxplot of RS calculated by four compared algorithms for both positive (labelled as 1) and negative (labelled as 0) samples

The utility of Neo-intline was firstly applied to detect the human neoantigens and compared with current state-of-the-art peers. Here, we selected three cutting-edge and widely used MHC-I neoantigen prediction pipelines including MuPeXi,^24^ Neopepsee,^25^ and pTuneos^26^ for comparison. Meanwhile, seven clinical samples including three melanoma patients,^18^ three gastrointestinal (GI) patients,^19^ and one breast cancer (BRCA) patient^20^ were derived as benchmark datasets. The validation was performed on the clinically validated peptides and the historically validated dataset derived from IEDB.^27^ The validation process was evaluated as Fig. 1 illustrated, which includes ranking the list of all possible peptides predicted by each approach (Fig. 1a), mapping the experimentally validated peptides in IEDB (Fig. 1b) and deriving the overlapped peptides (Fig. 1c) as the benchmark validation dataset for model comparison. Two parameters of RCS and RS were adopted to evaluate the performance of four peers. Detailed description of evaluation parameters was described in Methods part.

According to RCS, Neo-intline could win the best performance in five out of seven samples (sample ID: 3998, 3784, 3903, 4069 and 4136), followed by neopepsee for two samples (sample ID: 3995 and 4032) (Fig. 1d). Meanwhile, we noticed that Neo-intline could outperform three other methods on all the melanoma cases and BRCA cases but performed worse than others in all GI cases (Fig. 1d). These results suggested that Neo-intline might be more suitable for melanoma, and the prediction of neoantigens for different tumours may require different parameters for *in-silico* modelling. Moreover, the results of RS showed that Neo-intline could achieve the lowest RS of 0.43 for positive samples, followed by 0.45 for pTuneos, 0.48 for MuPeXI and 0.74 for neopepsee (Fig. 1e), which means Neo-intline could achieve smaller RS for positive samples than other approaches. Further, results showed that neopepsee can only detect 14 out of 34 positive samples and 5 out of 19 negative samples, which makes it difficult to distinguish between RS for positive samples and negative samples with both median value of 1 (Fig. 1f). Meanwhile, the median value of positive samples was 0.39, 0.30 and 0.37 for Neo-intline, MuPeXI and pTuneos, respectively. Among them, pTuneos only provided 11 different scores for over a thousand of peptides (from 0 to 1 with an interval of 0.1), which makes multiple peptides achieve the same ranking score. Thus, considering the median value of negative samples predicted by pTuneos was also 0.37, it is difficult to distinguish positive samples from negative ones (Fig. 1f). The median value of negative samples was 0.64 for Neo-intline and 0.92 for MuPeXI, respectively, which showed that those two methods could distinguish the positive samples and negative ones by ranking score.

In general, through the evaluation parameters of RCS and RS, Neo-intline could outperform other state-of-the-art tools at sample level (RCS won 5 out of 7 samples) and melanoma level. The good performance of Neo-intline might benefit from the *in-silico* design, which considers all the possible processes of peptide presentations. For example, the peptide of RILLVAASY that defined as negative sample,^19^ while only Neo-intline provided the RS of this peptide below the median RS of negative ones. This might be caused by the TAP process of Neo-intline, in which RILLVAASY achieves the logarithm IC50 value of 4.20, higher than the averaged logarithm IC50 value of 3.06.

### Model comparison on the TESLA community dataset and literature dataset

To further evaluate the prediction performance of Neo-intline compared with current available state-of-the-art peers, we introduced the widely used TESLA community dataset^21^ as the benchmark dataset. Here, three recently published neoantigen prediction tools of DeepNeo,^28^ Seq2Neo,^29^ and TSNAD v2.0^30^ were selected for performance comparison.

The TESLA community dataset contains 5 subjects with 3 melanoma patients and 2 non-small cell lung cancer (NSCLC) patients, which includes 535 validated peptides with 34 positive ones (Supplementary Data 1). The prediction performance was compared by evaluation parameters of AUC value, sensitivity, specificity and balanced accuracy. As illustrated in Supplementary Fig. 1a, Neo-intline could achieve the best AUC value of 0.6731, followed by 0.6357 for Seq2Neo, 0.5557 for DeepNeo and 0.4879 for TSNAD v2.0. For other parameters, Neo-intline could achieve the best-balanced accuracy of 0.6504, which outperformed the other three tools (Table 1). Note that, Seq2Neo could achieve the best sensitivity (0.7941) and TSNAD v2.0 could achieve the best specificity (0.9601) but with consequences of losing the performance on specificity (0.499) and sensitivity (0.1176), respectively.

**Table 1:** Prediction performance of AUC, sensitivity (sen), specificity (spe) and balanced accuracy (BA) for four tools

TESLA dataset	AUC	Cutoff	Sen	Spe	BA
Seq2Neo	0.6357	0.6178	**0.7941**	0.4990	0.6465
TSNAD v2.0	0.4869	0.8977	0.1176	**0.9600**	0.5388
DeepNeo	0.5557	0.7112	0.3235	0.8722	0.5978
Neo-intline	**0.6731**	0.0018	0.5882	0.7125	**0.6504**
Literature dataset	AUC	Cutoff	Sen	Spe	BA
Seq2Neo	0.5376	0.9275	0.5	0.6471	0.5735
TSNAD v2.0	0.5267	0.9897	0.5556	0.6471	0.6013
DeepNeo	0.6176	0.5782	0.6111	**0.6912**	0.6511
Neo-intline	**0.6944**	0.0003	**0.8333**	0.6029	**0.7181**

Bold values represent that Neo-intline achieves the best performance among all compared peers

Considering that the TESLA community dataset contains two different cancers, we separate them for further investigation. The melanoma dataset contains three patients, including 302 validated peptides with 26 positive ones. The NSCLC dataset contains two patients, including 233 validated peptides with 8 positive ones. The performance on melanoma illustrated the good performance in Neo-intline with AUC value of 0.7028, which could significantly outperform all other thee with the highest AUC value of 0.6311 for Seq2Ne. Meanwhile, the performance on NSCLC is relatively low for Neo-intline, which the AUC value is only 0.5325, lower than those of 0.6353 for TSNAD v2.0, 0.6283 for Seq2Neo and 0.5672 for DeepNeo.

Besides the TESLA community dataset, we also involved two experimentally validated datasets from previously published literature of chronic lymphocytic leukemia and melanoma patients for comparison.^22,23^ Considering those two datasets are small, we mixed them together for validation, which involved 86 validated peptides with 18 positive ones. Results showed that Neo-intline could achieve the best AUC value of 0.6944, which outperformed all other three (Supplementary Fig. 1b). Besides AUC value, Neo-intline could achieve the best sensitivity (0.8333) and balanced accuracy (0.7181), and comparable specificity (0.6029) among all four validated tools (Table 1).

### Successfully designed MHC-I and MHC-II neoantigens for mice melanoma

Above results illustrated that Neo-intline could outperform other state-of-the-art tools on sample level (RCS won 5 out of 7 samples) and melanoma level for both TESLA community data and literature datasets. Thus, we further test the pipeline of Neo-intline on real melanoma neoantigen vaccine design in mouse. After the pipeline of Neo-intline (*see Methods and Materials*), 32 MHC-I peptides (Supplementary Table 1) and 147 MHC-II peptides (Supplementary Table 2) were derived from mouse melanoma model. Next-generation sequencing data can be found in Supplementary Data 2 and Supplementary Data 3. The top 10 peptides for MHC-I (MHCI) and the top 10 peptides for MHC-II (MHCII) were selected to generate the melanoma neoantigen vaccine.

The predicted neoantigen probability scores (NPSs) for MHC-I peptides ranged from 2.67e^−4^ to 31.26, and the 10^th^ ranking candidate achieved an NPS score of 0.248, which is 928 times that of the 32^nd^ ranking candidate. Additionally, the final NPS for MHC-II peptides ranged from 2.77e^−7^ to 12.43, and the 10^th^ ranking candidate achieved an NPS score of 1.014, which is over 3 million times that of the 147^th^ ranking candidate (2.77e^−7^). Interestingly, several neoantigens occupying the top rankings in our study were previously proven to be able to drive therapeutic immune responses for cancer treatment. For example, the Gly795Ala mutation on Eef2 with the core peptide of LPVNESFAF (ranked 11 in Supplementary Table 1) and the Phe835Val mutation on Actn4 with the core peptide of VTFQAFIDV (rank 21 in Supplementary Table 1) for MHC-I were proven to be the functional neoantigens for melanoma.^17^ For MHC-II, 10 peptides were previously detected as neoantigens in Sebastian’s work,^17^ and were ranked from rank 28 to rank 122 among 147 MHC-II peptides in Supplementary Table 2. Among them, three were ranked within the top 50, including Tyr382His mutations on Gene Tm9sf3 with a core peptide of HHASRAIPFGTMVAV (rank 28), Asp314Asn mutations on Gene Cpsf3I with a core peptide of FDRTFANNPGPMVVF (rank 34), and Val602Ala mutations on Gene Ddx23 with a core peptide of TAMFTATMPPAVERL (rank 40). These peptides were proven to be efficient for tumour control and improving survival time in individuals with B16F10 melanoma. Moreover, we generated an epitope pool which included 9,215 previous experimentally validated Mus musculus peptides from IEDB or published articles. By 100% sequence identity searching through the epitope pool, six (MHC-I-1, 2, 6, 11, 19, and 21) out of top 21 MHC-I peptides and three (MHC-II-6, 16, 20) out of top 20 MHC-I peptides were detected as previously validated peptides (Supplementary Table 3), which illustrated the good performance of Neo-intline to serve the purpose for neoantigens screening.

Meanwhile, we adopted three cutting-edge tools including PMTnet,^31^ PanPep,^32^ and DLpTCR,^33^ to predict the peptide-CDR3 binding ability for top peptides (Supplementary Fig. 2). The TCR beta sequences of Mus musculus were derived from previous study,^34^ which included 85 identical CDR3 sequences. Further, we define a new probability score based on the prediction of three tools, in which peptide-TCR pairs can be scored as 1 (predicted as positive in all three methods), 0.75 (predicted as positive in two methods), 0.25 (predicted as positive in one method), and 0 (predicted as negative in all three methods). Then, for each peptide, the T-cell interaction score was defined as the percentage of positive CDR numbers over the pair score (Supplementary Fig. 2). Interestingly, by taking the 0.75 as the threshold for pair score, MHC-I-6 and MHC-I-19, which were experimentally validated before, could achieve the top two T-cell interaction scores that over 0.7. Moreover, all six experimentally validated peptides could achieve the T-cell interaction score over 0.5. By taking the value of MHC-I-1 as baseline, 6 out of top 10 MHC-I peptides may have real immunogenicity and hold the potential to elicit T-cell response, including 1, 2, 3, 6, 7, and 10, but 4 may probably fail (Supplementary Fig. 2a).

Similarly, we also validated the MHC-II peptides. Considering the MHC-II-6 was involved in the 55-mer peptide containing the mutation site, but not the previously predicted 15-mer core, we use the experimentally identified 15-mer core as the positive control for evaluation (Supplementary Fig. 2b). Results showed that peptide GRYFLKSSSATETMH derived from MHC-II-6 could achieve a relatively high T-cell interaction score of 0.4. Moreover, by setting the TIS of MHC-II-20 as a baseline, we found that MHC-II-5 could be considered as potential neoantigens that could elicit T-cell response (Supplementary Fig. 2b).

### In-vitro and In-vivo validation of neoantigens for melanoma

**Fig. 2:**
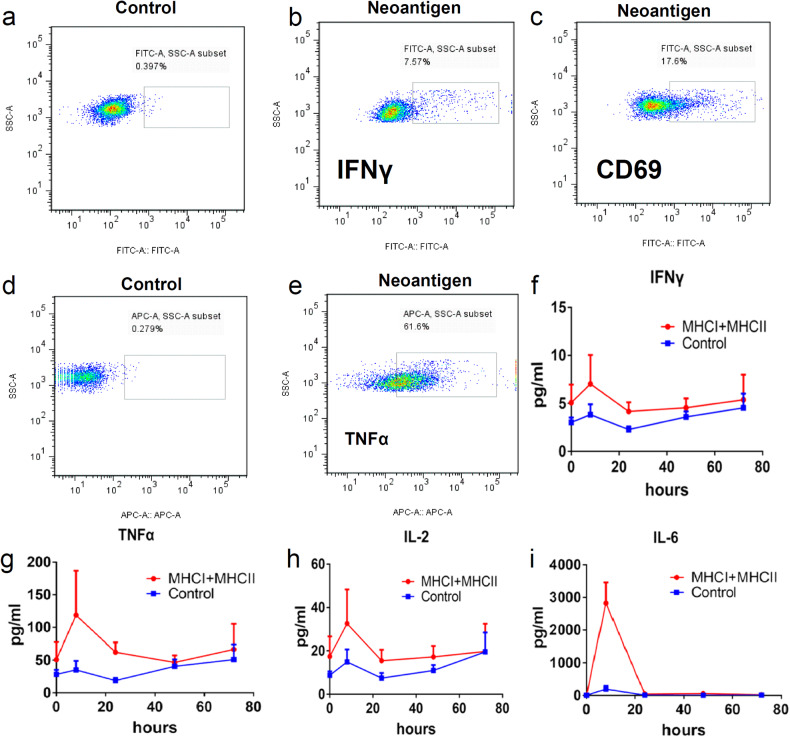
In-vitro validation of Neoantigen peptides. **a**–**e** In-vitro validation between neoantigen groups and control groups. IFN-γ and CD69 were detected by fluorescein isothiocyanate isomer (FITC). **a** Control group of IFN-γ and CD69. **b** Validation group of IFN-γ. **c** Validation group of CD69. **d** Control group of TNF-α. **e** Validation group of TNF-α. **f**–**i** Content of IFN-γ, TNF-α, IL-2 and IL-6 from peripheral blood in the neoantigen group and control groups at 0 h, 8 h, 24 h, 48 h and 72 h. **f** Content of IFN-γ. **g** Content of TNF-α. **h** Content of IL-2. **i** Content of IL-6

Experimentally, we first evaluated the ability of MHCI and MHCII to stimulate the immune response through an in-vitro incubation test with the T-cell repertoire. The fluorescent staining of fluorescein isothiocyanate isomer (FITC) and allophycocyanin (APC) showed that the neoantigens could significantly stimulate the activation of T cells after antigen presentation through the indicators of two cytokines (IFN-γ and TNF-α) and one cell biomarker of CD69 (Fig. 2a–e). Moreover, we measured the content of important cytokines, including IFN-γ, TNF-α, IL-2 and IL-6, in-vivo (Fig. 2f–i). Eight hours after vaccination, the content of four cytokines in peripheral blood peaked, and was at least 2 times higher than that in the control group (IFN-γ), and then started to decrease (Fig. 2f). The contents of IFN-γ, TNF-α and IL-2 remained higher than those in the control groups until 48 h (Fig. 2f–h). The levels of all four cytokines decreased to those of the control group at 72 h.

**Fig. 3:**
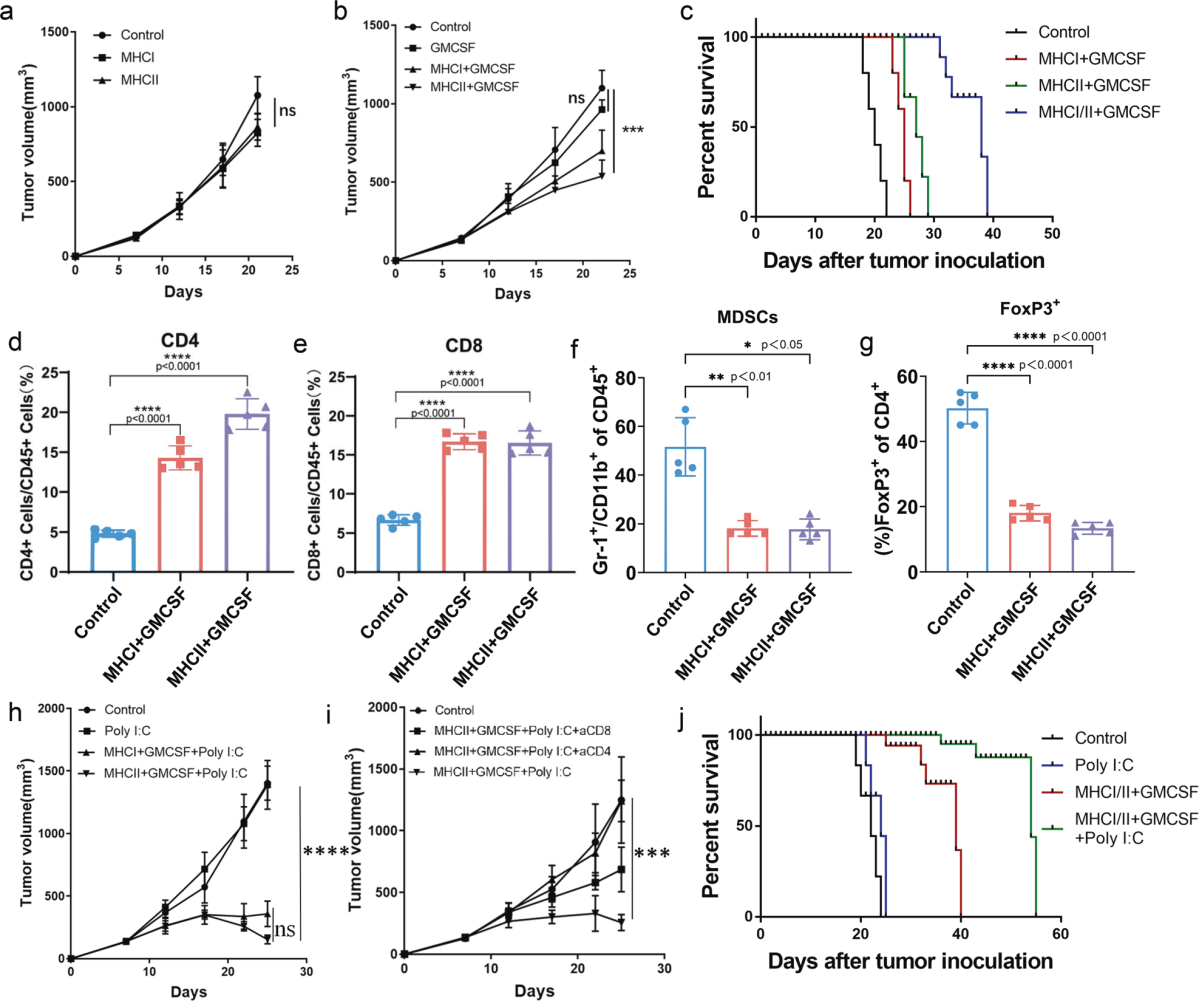
In-vivo validation on C57BL/6 mice. **a** Tumour volumes for C57BL/6 mice after using MHCI and MHCII as monotherapy. **b** Tumour volumes for C57BL/6 mice after using GMCSF as monotherapy or adjuvant with neoantigen vaccine. **c** Survival analysis of single neoantigen therapy and combined neoantigen therapy. **d** Percentage of CD4^+^ T cells among CD45^+^ lymphocytes on neoantigen combined with GMCSF. **e** Percentage of CD8^+^ T cells among CD45^+^ lymphocytes on neoantigen combined with GMCSF. **f** Percentage of MDSCs among CD45^+^ lymphocytes on neoantigen combined with GMCSF. **g** Percentage of FoxP3^+^ T-cells among CD4^+^ T cells on neoantigen combined with GMCSF. **h** Tumour volumes for C57BL/6 mice after treatment with Poly I:C, MHCI-GMCSF-Poly I:C and MHCII-GMCSF-Poly I:C. **i** Tumour volumes for C57BL/6 mice after treatment with MHCII-GMCSF-Poly I:C, MHCII-GMCSF-Poly I:C + aCD8, and MHCII-GMCSF-Poly I:C + aCD4. **j** Survival analysis of different therapies, including monotherapy of Poly I:C, combined therapy of MHCI/II-GMCSF, and combined therapy of MHCI/II-GMCSF-Poly I:C

Next, we used C57BL/6 mice to examine the efficiency of the neoantigen vaccine in model organisms. Firstly, we counted the tumour volume of each mouse from Day 0 (the day to split groups) to Day 22 (Fig. 3a–c). The tumour volumes decreased by 15% to 18% on Day 22 by using MHCI and MHCII (Fig. 3a). The granulocyte-macrophage colony-stimulating factor (GMCSF) monotherapy could decrease 13.6% of the tumour volume on Day 22, which is lower than MHCI and MHCII (Fig. 3b). The combination of neoantigen and GMCSF could achieve better performance, which could decrease 36.3% of the tumour volume (MHCI + GMCSF) and 45.5% of the tumour volume (MHCII + GMCSF) on Day 22 (Fig. 3b). The survival analysis illustrated that three treatment groups performed better than the control group (half death after 20 days) and the best performance occurred in the combination therapy group of neoantigen and GMCSF (MHCI/II + GMCSF), in which over 50% of the mice survived after 38 days (Fig. 3c). Further, we calculated the percentage of CD4^+^ and CD8^+^ T cells among CD45^+^ lymphocytes to evaluate the recruitment of functional T cells. Compared with the control, the percentage of CD4^+^ T cells increased approximately threefold by MHCI-GMCSF and above fourfold by MHCII-GMCSF (Fig. 3d). Additionally, both MHCI-GMCSF and MHCII-GMCSF increased the CD8^+^ T-cell percentage by ~2.5 times compared with the control group (Fig. 3e). Additionally, we evaluated two immune inhibition markers of Myeloid-derived suppressor cells (MDSCs) and Forkhead box protein P3 (FoxP3). The results showed that both MHCI-GMCSF and MHCII-GMCSF could significantly decrease the percentage of MDSCs (Fig. 3f) and FoxP3^+^ T-cells (Fig. 3g) compared with the control group.

In addition, we added polyinosinic-polycytidylic acid (Poly I:C), to the neoantigen vaccine as a combination therapy. Compared with the control group, monotherapy with Poly I:C illustrated no antitumour effects (Fig. 3h). The combinational therapy of MHCI-GMCSF and MHCII-GMCSF with Poly I:C significantly reduced the tumour volume after 17 days (Fig. 3h). On Day 22, the average tumour volume of the MHCII-GMCSF-Poly I:C treatment group was only 18.2% (mm^3^) of that of the control group (over 1400 mm^3^), which illustrated its potential treatment effect. Considering the great performance of MHCII-GMCSF-Poly I:C in reducing tumour volume, we evaluated the action model on tumour inhibition. After adding anti-mouse-CD8 (aCD8) antibody and anti-mouse-CD4 (aCD4) antibody to block the CD4 and CD8 protein on T cells, only the aCD8 group and MHCII-GMCSF-Poly I:C treatment group illustrated the ability to delay tumour growth, and only the MHCII-GMCSF-Poly I:C treatment group showed a decrease in tumour volume after 22 days (Fig. 3h). The tumour volumes of aCD4 group remained the same as those in the control group on Day 24. This phenomenon revealed that the function of MHCII-GMCSF-Poly I:C was mainly to stimulate the MHC-II-mediated T-cell immune response by recruiting and activating CD4^+^ T cells. Moreover, MHCII-GMCSF-Poly I:C could also recruit CD8^+^ T cells and stimulate the MHC-I-mediated T-cell immune response at a certain level (Fig. 3i). This might be caused by the cross-reactive immune response between MHC-I peptides and MHC-II peptides. Moreover, the survival analysis showed that the therapy group of MHCI/II-GMCSF-Poly I:C could significantly increase the survival rate compared with control group and other two therapy groups (Fig. 3j).

### Neoantigen vaccines combined with immune checkpoint inhibitors could enhance the antitumour effect in mice

In addition to broad-spectrum immune stimulant of Poly I:C, we also evaluated the performance of neoantigen vaccines combined with immune checkpoint inhibitors (ICIs) of mouse PD-1 antibody (mPD1) and mouse PD-L1 antibody (mPDL1). Here, we provide a novel perspective to evaluate whether the combination of *in-silico*-designed neoantigen vaccines and ICIs could increase the treatment effect.

**Fig. 4:**
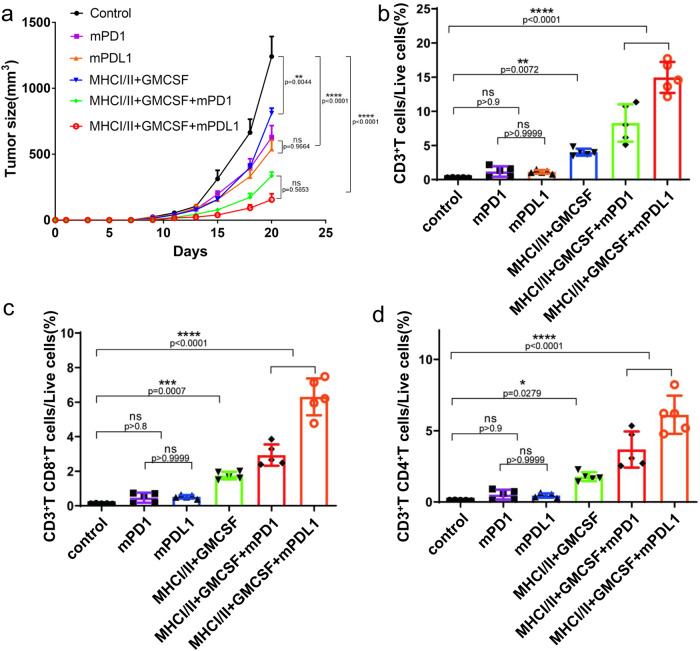
Effectiveness of neoantigen vaccine, immune checkpoint blockade and combinational therapy in C57BL/6 mice. **a** Tumour volumes of the control and five treatment groups. **b** Percentage of CD3^+^ T cells among all live cells in the control and five treatment groups. **c** Percentage of CD8^+^ T cells among all live cells in the control and five treatment groups. **d** Percentage of CD4^+^ T cells among all live cells in the control and five treatment groups

The results showed that on Day 20, the average tumour size of the control group was over 1200 mm.^3^ The tumour volume showed that the best treatment effects occurred in the MHCI/II-GMCSF-mPDL1 group, followed by treatment with MHCI/II-GMCSF-mPD1, mPDL1, mPD1 and MHCI/II-GMCSF (Fig. 4a and Supplementary Fig. 3). For the two neoantigen-ICI combination therapies, the tumour sizes were <500 mm^3^ on Day 20, which illustrated a significant effect (*P* < 0.0001) on the deceleration of tumour growth and higher than the other three therapies (Fig. 4a). Meanwhile, we evaluated the percentage of CD3^+^ T cells (Fig. 4b), CD8^+^ T cells (Fig. 4c), and CD4^+^ T cells (Fig. 4d) among all live cells. Results showed that control and ICI groups showed no statistical significance, while neoantigen-involving groups had a significantly increased number of CD3^+^ T cells, CD8^+^ T cells and CD4^+^ T cells (*P* < 0.001). The combined therapy of MHCI/II-GMCSF-mPDL1 illustrated the best treatment ability, which illustrated the potential synergistic effect on tumour suppression. The above results illustrated that the combination of the neoantigen vaccine and mPD1/mPDL1 antibody displayed a potential synergistic effect on the antitumour response, resulting in an additional effect on tumour suppression compared with the single treatment of the neoantigen vaccine and immune checkpoint blockade.

## Discussion

Neoantigen vaccines illustrated strong anticancer activities in multiple cancers, while not all neoantigen vaccines could provide the expected effects. To date, hundreds of clinical trials based on neoantigen vaccines have been conducted globally. Nevertheless, only a few treatments based on neoantigen vaccines show clinical benefit for tumour patients.^12,13,35^ Neoantigen therapy still shows uncertain efficacy for individual patients, leading low success rate in clinical trials.^9^ The main principle of the neoantigen vaccine is to detect the potential T-cell epitopes that have been produced by tumour mutation. Then, a neoantigen-based therapeutic vaccine can be constructed to induce cellular immunity. At present, the screening of cancer-specific neoantigens is dependent on multi-omics data and bioinformatics prediction. Although the current benchmark methods of T-cell epitope prediction can reach a high accuracy, only ~20% of the neoantigens are proven to be able to activate cellular immunity.^17^ The gap between such high prediction accuracy and low clinical effects is likely due to the complex immune presentation processes.

Usually, the bioinformatics prediction of neoantigen only focuses on the interaction between MHC-I and peptide. Some studies also consider the binding affinity between MHC-I-peptide and T-cell receptors but ignore other important steps, such as the hydrolysis of proteasome and the presentation of TAP.^25,26^ It’s possible that a potential antigenic peptide has a strong bind affinity with MHC-I molecule, but cannot be produced by proteasome or transported by TAP, uncapable of being presented to the cell surface. Therefore, the current bioinformatics prediction pipeline, which only focuses on MHC-I binding affinity, has certain limitations. This means that the gap between the high prediction accuracy of the benchmark method and the real clinical effectiveness may be due to the failure of the designed antigenic peptide to be presented. What’s more, early research on neoantigen prediction mainly focused on the design of MHC-I epitopes. However, recent studies have shown that the mutated MHC-II epitopes could also cause therapeutic anticancer immune responses. For example, Sebastian’s work showed that the majority of the immunogenic mutanome is recognized by CD4^+^ T cells.^17^ MHC-II epitope-based neoantigen vaccines may have strong anticancer activity. Thus, besides the multiple steps of MHC-I epitope presentation, the pipeline of neoantigen screening should also consider MHC-II neoantigens.

Therefore, considering the gaps between the *in-silico* design pipeline and clinical usage, we proposed Neo-intline, a neoantigen prediction pipeline that can be used as a benchmark for neoantigen vaccine design. This pipeline not only incorporates the design of both MHC-I and MHC-II neoantigens but also refines the presentation processes of the T-cell epitopes. Beginning from the mutations obtained through WGS, pipeline can predict the possibility of each mutation-containing peptide (MCP) from presentation to finally recognition by T cells. To achieve that, we fully considered the presentation processes of both MHC-I and MHC-II peptides and designed two scores, which can be used to rank and select the potential therapeutic MCPs for synthesis. The first validation *in-silico* on clinical trial data and TESLA community data showed the good performance of Neo-intline, especially on melanoma compared with current state-of-the-art peers. Moreover, we performed in-vitro and in-vivo validation for melanoma on C57BL/6 mice model. It should be noted that some of the antigenic peptides selected in previous studies were in our final candidate list but were not ranked in the top 10. The previous MHC-I neoantigens ranked from 11 to 21 among our list of 32 peptides. Previously detected MHC-II peptides were ranked from 28 to 122 among the list of 147 peptides. In this study, we screened the top 10 MCPs for MHC-I and MHC-II for subsequent experimental validation.

Through both in-vitro and in-vivo experiments, we found that the simple therapy of MHC-I neoantigen and MHC-II neoantigen vaccines could trigger T-cell immunity and illustrate useful but not significant anticancer effects in C57BL/6 melanoma mice. The combination of neoantigen vaccine with GM-CSF, Poly I:C and immune checkpoint inhibitors of PD1/PDL1 antibody could significantly improve the therapeutic effect of neoantigen peptides. Additionally, in different combination therapies, other ingredients cannot activate cellular immunity, while neoantigen peptides can recruit T cells, release cytokines, and induce subsequent T-cell-mediated cellular immunity. Notably, MHC-I peptides can also cause CD4^+^ T-cell-mediated cellular immunity, and the MHC-II peptides can cause CD8^+^ T-cell-mediated cellular immunity, which may be due to the cross-reaction between the MHC-I peptides and MHC-II peptides. These results showed that Neo-intline can accurately predict neoantigen peptides that could trigger cellular immunity through the immune presentation system and recruit functional T cells. Additionally, in clinical treatment, the neoantigen vaccine can be combined with other non-T-cell-targeted drugs, such as the broad-spectrum immune enhancement agents GM-CSF and Poly I:C and specific immune checkpoint inhibitors PD1 antibody and PDL1 antibody, to achieve better immunotherapeutic effects.

In general, we presented Neo-intline, the pipeline integrates the processes of T-cell immune presentations. Accepting sequence data as input, Neo-intline comprehensively considered all the possible processes in T-cell epitope presentation, including not only the proteasomal hydrolysis, TAP transport, MHC-I binding and TCR recognition steps in class I peptide presentation, and more importantly, took class II peptide presentation into account, therefore the high immunogenicity of the predicted peptides both in *in-silico*, in-vitro and in-vivo validation. The novelty and impact of Neo-intline reals in three levels: (1) introducing the idea of obtaining more accurate neoantigens by fully considering all the known steps in the T-cell epitope presentation processes, (2) providing *in-silico*, in vitro and in vivo validation to prove the peptides designed by Neo-intline is capable to stimulate T-cell immunity, (3) testing the therapeutic potential to combine the neoantigens with adjuvant and immune checkpoint inhibitors for melanoma treatment.

There are several limitations in this study to be considered when interpreting the results. The usage of Neo-intline could be influenced by the applicability and accuracy of the algorithms integrated in the pipeline. Note that, the current prediction tools may not fully consider the exact biological processes, for example, the proteasome cleavage may produce spliced peptides, therefore generating T epitopes different from exogenous protein sequences,^36^ which is not taken into account by currently used algorithm. Besides, in this study, we only considered the combination of neoantigen therapy with PD-1/PD-L1 antibodies. Other immune checkpoint inhibitors such as CTLA-4 antibody or other anti-cancer drugs may also be considered for the neoantigen-based combination therapy. In addition, we only provide the *in-silico* validation on human neoantigen detection, in-vitro and in-vivo validation on C57BL/6 mice model. The real clinical utility is yet to be verified by the subsequent experiments and clinical trials.

## Materials and methods

### Pipeline design of Neo-intline

**Fig. 5:**
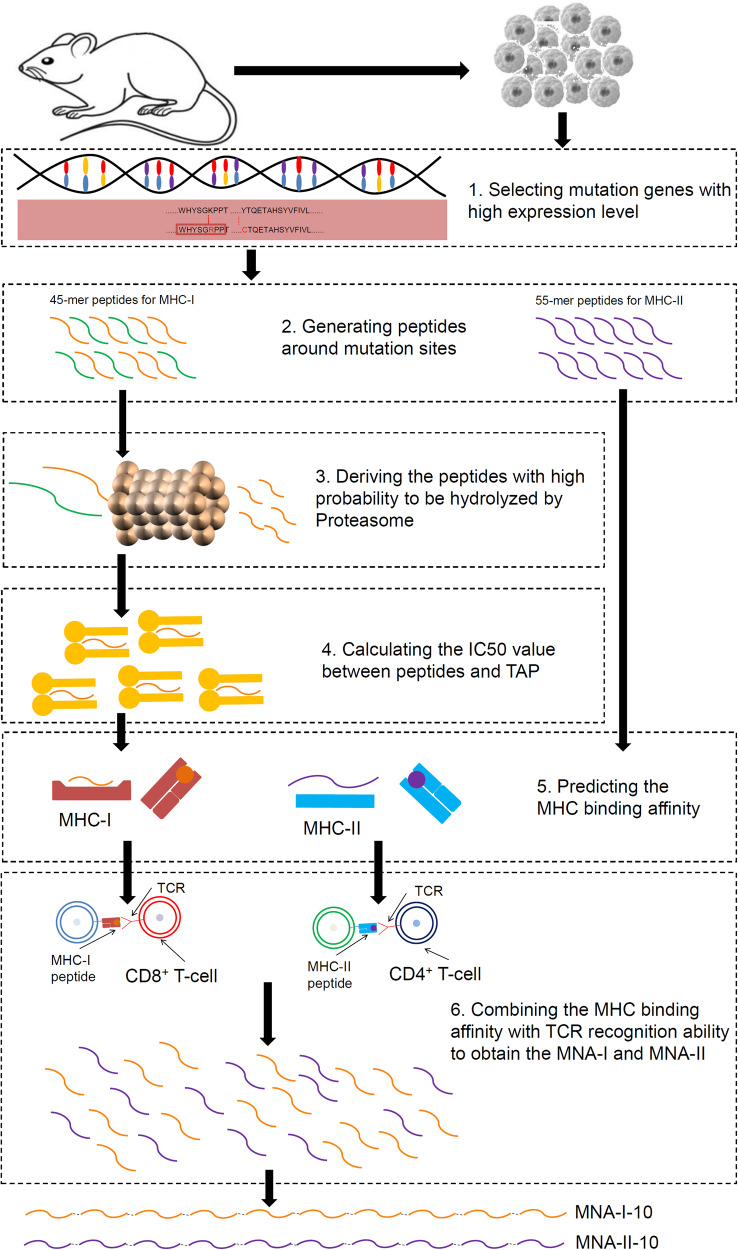
Workflow for pipeline Neo-intline. The NGS data of melanoma samples derived from C57BL/6 mice were aligned with the reference sequence to detect the mutation sites. After the six steps of Neo-intline, the top 10 ranking peptides for both MHC-I and MHC-II were linked with a flexible linker of GGGGSGGGGSGGGGS as two long peptides. The above peptides were expressed on a lentivirus vector for further evaluation and were marked as MNA-I-10 (MHCI) and MNA-II-10 (MHCII), respectively

The pipeline of Neo-intline is illustrated in Fig. 5. The main purpose of Neo-intline was to simulate the biological processes of T-cell epitope presentation, which included (1) selecting mutations with high expression levels, (2) generating 45-mer peptides for MHC-I and 55-mer peptides for MHC-II around the mutation sites, as MCPs, (3) deriving the peptides with a high possibility of being hydrolysed by the proteasome for MHC-I MCPs, (4) calculating the theoretical IC_50_ value between MCPs and TAP for MHC-I MCPs, (5) predicting the binding affinity between MCPs and two types of MHC, and (6) combining the above steps with the TCR recognition score to obtain the final ranking of both MHC-I neoantigens (MNA-I) and MHC-II neoantigens (MNA-II). Among the above, the in-vivo processing steps can be systemically simulated through Neo-intline. Detailed information for each step can be found below.

### Whole exome sequencing and transcriptome resequencing

The Whole Exome Sequencing was performed by a commercial provider (BGI-Tech). The DNA and RNA from B16F10 cells and DNA from the tail tissue of C57BL/6 mice were extracted in triplicate for sequencing. The bioinformatics analysis began from the sequencing data (raw data) generated on Illumina HiSeq2000 platform. Firstly, the adaptor sequence in the raw data was removed, and low-quality reads which have too many Ns or low base quality were discarded. Secondly, Burrows-Wheeler Aligner (BWA)^37^ was used to do the alignment. The reference genome uses the mouse genome mm10 (http://hgdownload.cse.ucsc.edu/goldenPath/mm10/bigZips/). Then, BWA can convert the sequence data into BAM format files. After that, the BAM format files were further processed, such as fixing mate information of the alignment, adding read group information, and removing duplicate reads caused by polymerase chain reaction (PCR). After these processes, the final BAM files used to do the variant calling got ready. Single Nucleotide Polymorphisms (SNPs) were called by Genome Analysis Toolkit (GATK).^38^ After that, some filters were applied to get more confident variant results. Then, the commercial provider (BGI-Tech) uses AnnoDB which is in-house to annotate the confident variant results. The final variants can feed to the downstream advanced analysis pipeline. Quality Control (QC) was present in the whole pipeline for the clean data, the alignment, and the called variant. In this process, we detected melanoma-associated somatic mutated genes from SNP analysis shown in Supplementary Data 2 and Supplementary Data 3, respectively.

The Transcriptome Resequencing was also performed by BGI-Tech, in which the raw data generated from the Illumina HiSeq platform was analysed through the standard pipeline. Samples were extracted from the mouse tumour cells B16F10 and tail tissue samples of C57BL/6 mice, which were sequenced in triplicate. Then, quality control was conducted by the following filter criteria: (1) Remove reads with adaptors; (2) Remove reads in which unknown bases (N) are >5%; (3) Remove low-quality reads (we define the low-quality read as the percentage of the base which quality is <15 is >20% in a read). Next, HISAT^39^ was used to map the clean reads to mm10 from http://hgdownload.cse.ucsc.edu/goldenPath/mm10/bigZips/. Finally, fragments Per Kilobase of the exon model per Million mapped fragments (FPKM) were obtained for each gene according to the sequencing reports.

### Peptide preparation

For each mutation at position *m*, a peptide ranging from position *m-n* to *m* + *n* was defined as the tumour-specific mutation peptide *P*_*1*_ *(m-n,m* + *n)*. The peptides derived from wild-type samples (no mutation) and tumour samples (mutation) were defined as ${P}_{{WT}}$ and ${P}_{M}$, respectively.

Here, for MHC-I, *n* = *22*, while for MHC-I, *n* = 27. Thus, 45-mer peptides for MHC-I and 55-mer peptides for MHC-II can be generated as mutation-containing peptides (MCPs). Note that if the mutation site nears the N-terminal or C-terminal, the peptide length may be <2n + *1*. Furthermore, core peptides of the 9-mer for MHC-I and 15-mer for MHC-II involving the mutation sites were derived from MCPs, which included 4,392 peptides for MHC-I and 7,302 peptides for MHC-II.

After peptide preparation, we prepared the *in-silico* simulation mode, which includes (1) preselection according to the gene expression level of transcripts per kilobase of exon model per million mapped reads (TPM); (2) derivation of the peptides with a high probability of being hydrolyzed by the proteasome, for MHC-I peptide only; (3) calculation of the IC_50_ value between peptides and the transporter associated with antigen processing (TAP), for MHC-I peptide only; (4) prediction of the MHC binding affinity between peptide and MHC molecules; and (5) combination of the scores of the above steps with the TCR recognition score to obtain the final neoantigen probability score (NPS). Then, both the MHC-I epitopes (MNA-I) and MHC-II epitopes (MNA-II) were ranked according to NPS.

### Preselection of peptides based on the expression level

The expression level of the mutant genes could influence the immunogenicity of neoantigens. Before peptide selection, the expression level should be considered as a filtering condition. From the sequence data, the fragments per kilobase of the exon model per million mapped fragments (FPKM) were obtained for each gene. Then, the FPKM score was transformed to transcripts per kilobase of exon model per million mapped reads (TPM). Next, the TPM score can be mapped to each peptide according to the mutation sites and the corresponding located genes. The TPM score for each MCP was defined using Formula (1):

1 $${TS}=\left\{\begin{array}{ccc}1, & {if} & {{TPM}}_{i} \,>\, 3\\ 0, & {if} & {{TPM}}_{i}\le 3\end{array}\right.$$

where TS represents the TPM Score, and for each peptide, only when the TPM of its located gene is >3 will the TS score be 1; otherwise, it is 0.

### Deriving the hydrolysis probability score of each peptide

The proteasome hydrolysis score of each peptide was predicted through Netchop^40^ with default parameters. The proteasome hydrolysis score (PHS) was defined using Formula (2):

2 $${PHS}=\left\{\begin{array}{ccccc}1, & {if} & {N}^{{\prime} }P \,>\, 0.7 & {or} & {C}^{{\prime} }P \,>\, 0.7\\ 0, & {if} & {N}^{{\prime} }P\le 0.7 & {and} & {C}^{{\prime} }P\le 0.7\end{array}\right.$$

Note that Netchop outputs two different possibility scores, including the N’ hydrolysis score and C’ hydrolysis score, and in this study, we defined that if the possibility score is at least over 0.7; this peptide will be presented in the next step.

### Predicting the TAP score of each peptide

Here, the TAP binding ability was predicted through Besser H’s work^41^ with default parameters. The output IC_50_ value was used as the TAP score (T). Notably, low IC50 values represent a high affinity and a higher likelihood of binding. Thus, the score T is negatively correlated with the potential to become neoantigen peptides. The TAP binding score (TBS) was defined as the logarithm value of the IC_50_ value in further calculations. The defined TAP score of IC50 was defined as TIC, which is defined as TIC = TBS + |TBS_min_ |.

### Tumour-specific MHC-I binding peptide prediction

For MHC-I epitope prediction, the binding affinity of all the 45-mer peptides, including both ${P}_{{WT}1}$ and ${P}_{M1}$, was predicted through NetMHCpan 4.0.^11^ The segment length of the T-cell epitope was set as the 9-mer, and mouse MHC alleles of H2-Dd, H2-Kd, H2-Ld, H2-Db, and H2-Kb were involved. Then, all of the 9-mer segments ${S}_{{WT}1}$ and ${S}_{M1}$ were split from ${P}_{{WT}1}$ and ${P}_{M1}$ using a 9-mer length sliding window, and the binding affinity between each segment and each MHC allele was predicted according to the default threshold. Only those ${S}_{M1}$ with MHC-I binding affinity <500 nM were selected as potential neoantigen candidates, and the affinity ratio ${\rm{A}}\left({S}_{1}\right)$ was calculated using Formula (3):

3 $${\rm{A}}\left({S}_{1}\right)=\frac{{Aff}({S}_{{WT}1})}{{Aff}({S}_{M1})}$$

where ${Aff}({S}_{{WT}1})$ refers to the affinity score of the segment S_1_ in ${P}_{{WT}1}$, and ${Aff}({S}_{M1})$ refers to the affinity score of the segment S_1_ in ${P}_{M1}$. Here, score ${\rm{A}}\left({S}_{1}\right)$ represents the magnification times of MHC-I binding caused by the mutation sites.

### Tumour-specific MHC-II binding peptide prediction

For MHC II binding peptides, the 55-mer peptides at corresponding positions in wild-type samples and tumour samples were marked as ${P}_{{WT}1}$ and ${P}_{M1}$, respectively. The binding affinity score of all the 55-mer peptides was predicted through NetMHCIIpan.^11,42^ Different from MHC-I, the segment length of MHC-II peptides was set as the 15-mer, and MHC alleles of H2-IAb and H2-IAd were selected. Then, all of the 15-mer segments ${S}_{{WT}2}$ and ${S}_{M2}$ were split from ${P}_{{WT}2}$ and ${P}_{M2}$ using a 15-mer length sliding window, and the binding affinity between each segment and each MHC allele was predicted according to the default threshold. Similarly, only those ${S}_{M2}$ with MHC-I binding affinity <500 nM were selected as potential neoantigen candidates, and the affinity ratio ${\rm{A}}\left({S}_{2}\right)$ was calculated using Formula (4):

4 $${\rm{A}}\left({S}_{2}\right)=\frac{{Aff}({S}_{{WT}2})}{{Aff}({S}_{M2})}$$

where ${Aff}({S}_{{WT}2})$ refers to the affinity score of the segment S_2_ in ${P}_{{WT}2}$, and ${Aff}({S}_{M2})$ refers to the affinity score of the segment S_2_ in ${P}_{M2}$. Here, score ${\rm{A}}\left({S}_{2}\right)$ represents the magnification times of MHC-II binding caused by the mutation sites.

### TCR recognition probability

To test the ability of the predicted MHC-binding peptide to be recognized by the TCR, the probability score of *R* was calculated. Typically, for a given neoantigen peptide *S*, the TCR-recognition score *R* represents the probability that S is recognized by the T-cell receptor repertoire. The value of *R* was estimated based on the evaluation of similarities between experimentally determined MHC-binding peptides and predicted peptides. First, the epitope peptides determined by the T-cell binding test were collected from the public database of the Immune Epitope Database and Analysis Resource (IEDB).^27^ The sequence similarity between predicted MHC binding peptides and IEDB-determined peptides was calculated by BLASTp using BLOSUM62 as the substitution matrix with a gap opening penalty of -11 and a gap extension penalty of -1. The sequence similarity score was defined as |s,e| in Formulas (5) and (6), and the T-cell recognition probability R was calculated.

5 $${\rm{R}}=Z{(k)}^{-1}\sum _{e\in IEDB}\exp [-k(a-|s,e|]$$

6 $${\rm{Z}}({\rm{k}})=1+\sum _{e\in IEDB}\exp [-k(a-|s,e|]$$

where R represents the probability score of T-cell recognition, *a* represents the horizontal displacement of the binding curve, and *k* is the slope of the curve at a. Here, *a* was set as 26, and $k$ was set as 1 according to a previous study.^5^

### Neoantigen probability score

For each of the target peptides, the final neoantigen probability score (NPS) for immunogenicity was calculated using Formula (7) for MHC-I binding peptides and Formula (8) for MHC-II binding peptides. The MHC-binding peptides with the top-ranking NPS were selected for further experimental validation.

7 $${{NPS}}_{{MHC}-I}={TS}\,\cdot\, {PHS}\,\cdot\, A\left({S}_{1}\right)\,\cdot\, R/{TIC}$$

8 $${{NPS}}_{{MHC}-2}={TS}\,\cdot\, A\left({S}_{2}\right)\,\cdot\, R$$

where ${{NPS}}_{{MHC}-I}$ represents the neoantigen probability score for MHC-I and ${{NPS}}_{{MHC}-2}$ represents the neoantigen probability score for MHC-II.

### Model comparison

The prediction of four approaches (Neo-intline, MuPeXi, Neopepsee, and pTuneos) will provide a list of all possible peptides in 8-mer, 9-mer, 10-mer and 11-mer, with the ranking score (Fig. 1a). Further, according to the 100% identity of peptides matched in the experimentally validated peptides from the above three papers and from IEDB^27^ (Fig. 1b), a total number of 53 peptides (Supplementary Table 4) were derived as the benchmark validation dataset for model comparison (Fig. 1c). We obtained the first rank values of matched peptides with a unique MHC allele. Then, the rank coverage score (RCS) from pTuneos was adopted as evaluation parameters, which was shown in formula (S1) and formula (S2) illustrated:^26^

9 $${\rm{RankCoverageScore}}=\frac{\sum _{n\in {negative}}{{rank}}_{n}}{T\times {num}(n)}\times {coverage}\left(n\right)-\frac{\sum _{p\in {positive}}{{rank}}_{p}}{T\times {num}\left(p\right)}\times {coverage}\left(p\right)$$

10 $${\rm{coverage}}\left({\rm{k}}\right)=\frac{\max ({{rank}}_{k})}{T}k\in \left(n,p\right)$$

The RCS ranges between -1 and 1, where larger RCS represents better ranking performance.

Moreover, we defined the ranking score (RS) for each peptide in the prediction list of all four tools, which was defined as the ranking of the corresponding peptides divided by the total number of all the peptides in the ranking list. The value range of RS is between 1/n (the total number in the ranking list) and 1 (ranked as the last one), where 1/n means top 1 ranking and 1 means the last ranking. In an ideal way, the positive samples should achieve lower RS than the negative ones. According to the above, the RS of all the experimentally validated positive peptides were calculated and the peptide didn’t been detected in each algorithm was marked as 1.

The prediction of Neo-intline compare with DeepNeo, Seq2Neo and TSNAD v2.0 were using the binary classification parameters including AUC value, balanced accuracy, sensitivity and specificity, which were given as formula (11) to (13) illustrated.

11 $${\rm{Sensitivity}}=\frac{{TP}}{{TP}+{FN}}$$

12 $${\rm{Specificity}}=\frac{{TN}}{{TN}+{FP}}$$

13 $${\rm{Balanced\; Accuracy}}=\frac{S{ensitivity}+S{pecificity}}{2}$$

Where TP stands for true positive samples, TN stands for true negative samples, FP stands for false positive samples and FN stands for false negative samples.

### Mouse models

All animal experiments were approved by the Animal Ethics Committee of Tongji University. Female C57BL/6 J mice (6–8 weeks old) were purchased from SLRC Laboratory Animal Co., Ltd. (Shanghai, China) and were housed in a pathogen-free animal facility at the experimental animal center. Mice were fed standard chow and provided with distilled water ad libitum. Sanitized cages with fresh bedding were provided weekly. After the experiments, the mice were anesthetized with pentobarbital sodium (60 mg/kg) and euthanized using 100 mg/kg sodium pentobarbital. Appropriate efforts were made to minimize animal suffering.

C57BL/6 J mice bearing a total of 2.5 ×10^5^ subcutaneous (s.c.) B16F10 tumour cells were randomly assigned to treatment groups (6–8 mice per group), with the mean tumour volume for each group being 100–150 mm^3^. Then, the DNA vector (neoantigen, MHCI or MHCII-GMCSF) was injected subcutaneously (sc.) at a dosage of 20 μg twice a week from Day 0. Tumour sizes were measured with a digital caliper every other day and calculated as length × width^2^ × 0.5.

For combination therapy, mice were s.c. injected with B16F10 tumour cells and i.v. injected on the same day with DNA vector (20 μg), alone or combined with anti-mPD-1 antibody (10 mg/kg), anti-mPD-L1 antibody (10 mg/kg) or Poly(I:C) (i.t. injected) 50 µg twice a week. Tumour growth was monitored every other day.

### Generation of BM-derived DCs

BM cells were flushed from femurs and plated in a 6-well plate at 2 × 10^6^ cells in 3–6 mL of medium comprised of RMPI-1640 (Gibco, Bleiswijk, The Netherlands) supplemented with 10% FBS (Gibco), 25 nM β-mercaptoethanol (Sigma), 100 U/mL penicillin (Eureco Pharma), 100 µg/mL streptomycin (Sigma) and 20 ng/mL GM-CSF (PeproTech, Hamburg, Germany) for 8 days, with a medium change on Day 4 and Day 7. Subsequently, nonadherent GMDCs were used for various assays.

### T-Cell priming assay

Top 10 ranking MHC-I peptides of 45-mer length (Supplementary Table 1) and Top 10 ranking MHC-II peptides of 55-mer length (Supplementary Table 2) were synthesized by the Sangon Biotech Co., Ltd. We concatenated the DNA sequences of each top 10 neoantigens together, with a GS-linker in between. For in-vitro T-cell priming assay, we added 50 μl of undiluted supernatant containing the expressed neoantigen to the plate, which was plated with both DC cells and T cells, the peptide concentration of neoantigen peptides is 10 μM. For in-vivo study, we selected a 20 μg DNA plasmid for administration. For in-vitro T-cell priming assays, 10^5^ DC cells were cocultured with OVA and neoantigen in serum-free RPMI medium for 3 h at 37 °C. T cells were isolated using biotinylated anti-CD3 antibodies, followed by enrichment with antibiotin magnetic beads (Miltenyi Biotec). Then, 1 × 10^6^ T cells were added to neoantigen-treated DCs for 4 h. CD86 and CD11C positive DCs were analysed by flow cytometry. TNF-α and IFN-γ produced during the priming of T cells were measured by flow cytometry.

### Analysis of tumour-infiltrating lymphocytes (TILs) and MDSCs

After treatment with the vaccine, tumours were collected and dissociated using a mouse tumour dissociation kit with a gentle MACS Octo Dissociator (Miltenyi Biotec) according to the manufacturer’s protocol. Tumour-infiltrating cells were analysed by flow cytometry. CD11b^+^Gr-1^+^ cells were labelled with APC-conjugated Gr-1 and FITC-conjugated CD11b antibodies.

Single-cell suspensions (10^6^ cells/100 μl) were preincubated with a purified rat anti-mouse CD16/CD32 monoclonal antibody (Fc block, clone 2.4 G, BD Biosciences) and then stained with one of the following fluorescently labelled antibodies at 4 °C for 30 min: anti-CD45-AF 700 (clone 30-F11), anti-CD3-APC (clone 145-2C11), anti-CD4-PE-Cy7 (clone RM4-5), anti-CD8-Percp-Cy5.5 (clone 53-6.7), anti-Foxp3-FITC (clone FJK-16s), anti-GR-1-APC (clone RB6-8C5), and anti-CD11b-FITC (clone M1/70) antibodies purchased from BD Biosciences.

### Supplementary information

Supplementary Materials for Neo-intline: integrated pipeline enables Neoantigen design through the in-silico presentation of T-cell epitope

Data S1

Data S2

Data S3

## Data Availability

All data and materials are presented in the main manuscript or supplementary materials and are available on request. Main algorithms of Neo-intline can be found in Github at: https://github.com/zoolie/neoantigenPre.

## References

[1] Sung H (2021). Global Cancer Statistics 2020: GLOBOCAN estimates of incidence and mortality worldwide for 36 cancers in 185 countries. Ca. Cancer J. Clin..

[2] Pich O (2022). The translational challenges of precision oncology. Cancer Cell..

[3] Hu Z (2018). Towards personalized, tumour-specific, therapeutic vaccines for cancer. Nat. Rev. Immunol..

[4] Vormehr M (2016). Mutanome directed cancer immunotherapy. Curr. Opin. Immunol..

[5] Balachandran VP (2017). Identification of unique neoantigen qualities in long-term survivors of pancreatic cancer. Nature.

[6] Ott PA (2017). An immunogenic personal neoantigen vaccine for patients with melanoma. Nature.

[7] Keskin DB (2019). Neoantigen vaccine generates intratumoral T cell responses in phase Ib glioblastoma trial. Nature.

[8] Schumacher TN (2015). Biomarkers in cancer immunotherapy. Cancer Cell..

[9] Blass E (2021). Advances in the development of personalized neoantigen-based therapeutic cancer vaccines. Nat. Rev. Clin. Oncol..

[10] Snyder A (2015). Immunogenic peptide discovery in cancer genomes. Curr. Opin. Genet. Dev..

[11] Reynisson B (2020). NetMHCpan-4.1 and NetMHCIIpan-4.0: improved predictions of MHC antigen presentation by concurrent motif deconvolution and integration of MS MHC eluted ligand data. Nucleic Acids Res..

[12] Hu Z (2021). Personal neoantigen vaccines induce persistent memory T cell responses and epitope spreading in patients with melanoma. Nat. Med..

[13] Guo Z (2022). Durable complete response to neoantigen-loaded dendritic-cell vaccine following anti-PD-1 therapy in metastatic gastric cancer. Npj. Precis. Oncol..

[14] Greene ER (2020). Understanding the 26S proteasome molecular machine from a structural and conformational dynamics perspective. Curr. Opin. Struct. Biol..

[15] Suh WK (1994). Interaction of MHC class I molecules with the transporter associated with antigen processing. Science.

[16] Luksza M (2017). A neoantigen fitness model predicts tumour response to checkpoint blockade immunotherapy. Nature.

[17] Kreiter S (2015). Mutant MHC class II epitopes drive therapeutic immune responses to cancer. Nature.

[18] Gros A (2016). Prospective identification of neoantigen-specific lymphocytes in the peripheral blood of melanoma patients. Nat. Med..

[19] Tran E (2015). Immunogenicity of somatic mutations in human gastrointestinal cancers. Science.

[20] Zacharakis N (2018). Immune recognition of somatic mutations leading to complete durable regression in metastatic breast cancer. Nat. Med..

[21] Wells DK (2020). Key parameters of tumor epitope immunogenicity revealed through a consortium approach improve neoantigen prediction. Cell.

[22] Rajasagi M (2014). Systematic identification of personal tumor-specific neoantigens in chronic lymphocytic leukemia. Blood.

[23] Stronen E (2016). Targeting of cancer neoantigens with donor-derived T cell receptor repertoires. Science.

[24] Bjerregaard AM (2017). MuPeXI: prediction of neo-epitopes from tumor sequencing data. Cancer Immunol. Immunother..

[25] Kim S (2018). Neopepsee: accurate genome-level prediction of neoantigens by harnessing sequence and amino acid immunogenicity information. Ann. Oncol..

[26] Zhou C (2019). pTuneos: prioritizing tumor neoantigens from next-generation sequencing data. Genome Med..

[27] Dhanda SK (2019). IEDB-AR: immune epitope database-analysis resource in 2019. Nucleic Acids Res..

[28] Kim JY (2023). DeepNeo: a webserver for predicting immunogenic neoantigens. Nucleic Acids Res..

[29] Diao, K. et al. Seq2Neo: A comprehensive pipeline for cancer neoantigen immunogenicity prediction. *Int. J. Mol. Sci*. **23**, 11624 (2022). 10.3390/ijms231911624 PMC9569519 36232923

[30] Zhou Z (2021). TSNAD v2.0: A one-stop software solution for tumor-specific neoantigen detection. Comput. Struct. Biotechnol. J..

[31] Lu T (2021). Deep learning-based prediction of the T cell receptor-antigen binding specificity. Nat. Mach. Intell..

[32] Yicheng G (2023). Pan-Peptide Meta Learning for T-cell receptor–antigen binding recognition. Nat. Mach. Intell..

[33] Xu, Z. et al. DLpTCR: an ensemble deep learning framework for predicting immunogenic peptide recognized by T cell receptor. *Brief. Bioinform*. **22**, bbab335 (2021). 10.1093/bib/bbab335 34415016

[34] Tickotsky N (2017). McPAS-TCR: a manually curated catalogue of pathology-associated T cell receptor sequences. Bioinformatics.

[35] Rubinsteyn A (2017). Computational Pipeline for the PGV-001 Neoantigen Vaccine Trial. Front. Immunol..

[36] Vigneron N (2019). Production of spliced peptides by the proteasome. Mol. Immunol..

[37] Li H (2009). Fast and accurate short read alignment with Burrows-Wheeler transform. Bioinformatics.

[38] McKenna A (2010). The Genome Analysis Toolkit: a MapReduce framework for analyzing next-generation DNA sequencing data. Genome Res..

[39] Kim D (2015). HISAT: a fast spliced aligner with low memory requirements. Nat. Methods.

[40] Kesmir C (2002). Prediction of proteasome cleavage motifs by neural networks. Protein Eng..

[41] Besser H (2018). Cross-modality deep learning-based prediction of TAP binding and naturally processed peptide. Immunogenetics.

[42] Karosiene E (2013). NetMHCIIpan-3.0, a common pan-specific MHC class II prediction method including all three human MHC class II isotypes, HLA-DR, HLA-DP and HLA-DQ. Immunogenetics.

