# Supplementary material for: Neo-intline: integrated pipeline enables neoantigen design through the in-silico presentation of T-cell epitope
Source: Signal Transduct Target Ther. 2023 Oct 18;8:397. doi: 10.1038/s41392-023-01644-9 (PMC10582007; doi:10.1038/s41392-023-01644-9)
Supplement: Supplementary file 1 — Supplementary Materials for Neo-intline: integrated pipeline enables Neoantigen design through the in-silico presentation of T-cell epitope [file 41392_2023_1644_MOESM1_ESM.docx]

Supplementary Materials for

**Neo-intline: integrated pipeline enables Neoantigen design through the *in-silico* presentation of T-cell epitope**

Bingyu Li^1,2,#^, Ping Jing^1,#^, Genghui Zheng^3, 4, #^, Chenyu Pi^1^, Lu Zhang^1^, Zuojing Yin^3^, Lijun Xu^1,2^, Jingxuan Qiu^5^, Hua Gu^1^, Tianyi Qiu^3,6,*^, Jianmin Fang^1*^

^1^ Laboratory of Molecular Medicine, Shanghai Key Laboratory of Signaling and Disease Research, School of Life Sciences and Technology, Tongji Hospital, Tongji University Suzhou Institute, Tongji University, Shanghai, China.

^2^ School of Basic Medical Sciences, Henan University of Science and Technology, Luoyang, Henan, China.

^3^ Institute of Clinical Science, Zhongshan Hospital, Fudan University, Shanghai, China.

^4^ Oden Institute for Computational Engineering and Sciences (ICES), University of Texas at Austin, Austin, Texas, United States.

^5^ School of Health Science and Engineering, University of Shanghai for Science and Technology, Shanghai, China.

^6^ Shanghai Institute of Infectious Disease and Biosecurity, Fudan University, Shanghai, 200032, China.

^#^ These authors contributed equally to this work.

*Correspondence should be addressed to Dr. Tianyi Qiu ([ty_qiu@126.com](mailto:ty_qiu@126.com)) or Dr. Jianmin Fang ([jfang@tongji.edu.cn](mailto:jfang@tongji.edu.cn))

**This PDF file includes:**

Figures. S1 to S3

Tables. S1 to S4

**Other Supplementary Materials for this manuscript include the following:**

Data S1 to S3


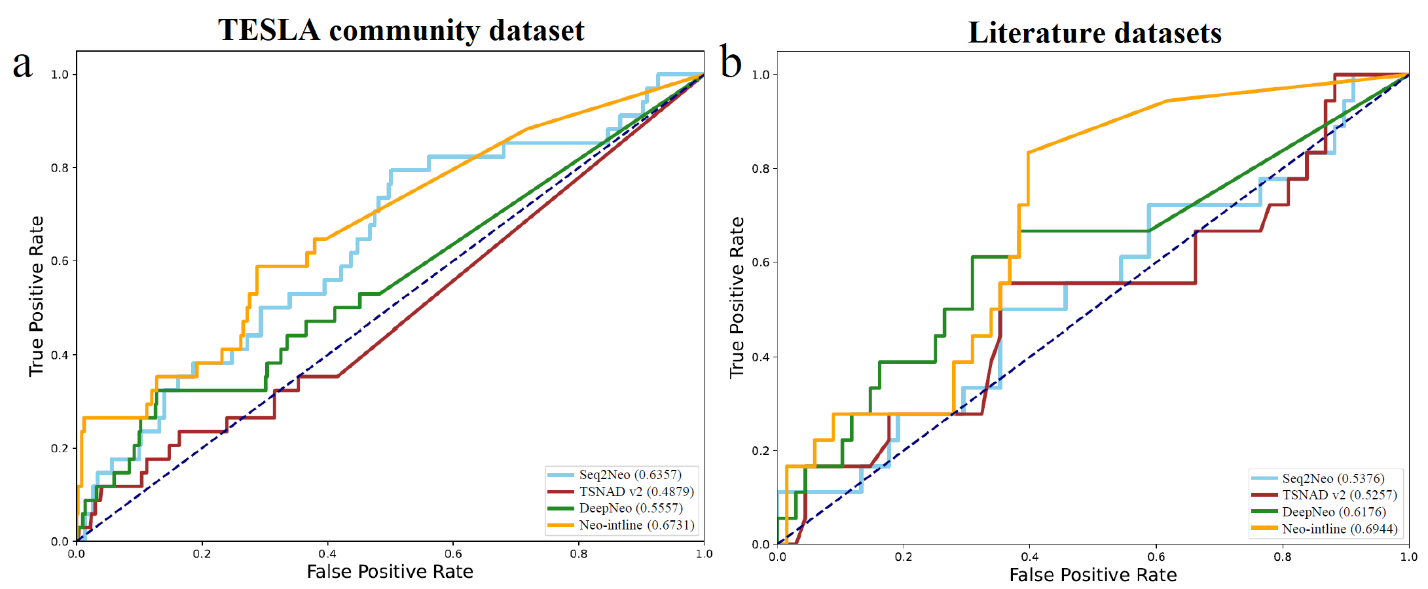
Figure. S1. ROC Curve in different models. a ROC curves of Seq2Neo, TSNAD v2.0, DeepNeo and Neo-intline on TESLA community dataset. b ROC curves of Seq2Neo, TSNAD v2.0, DeepNeo and Neo-intline on literature datasets.


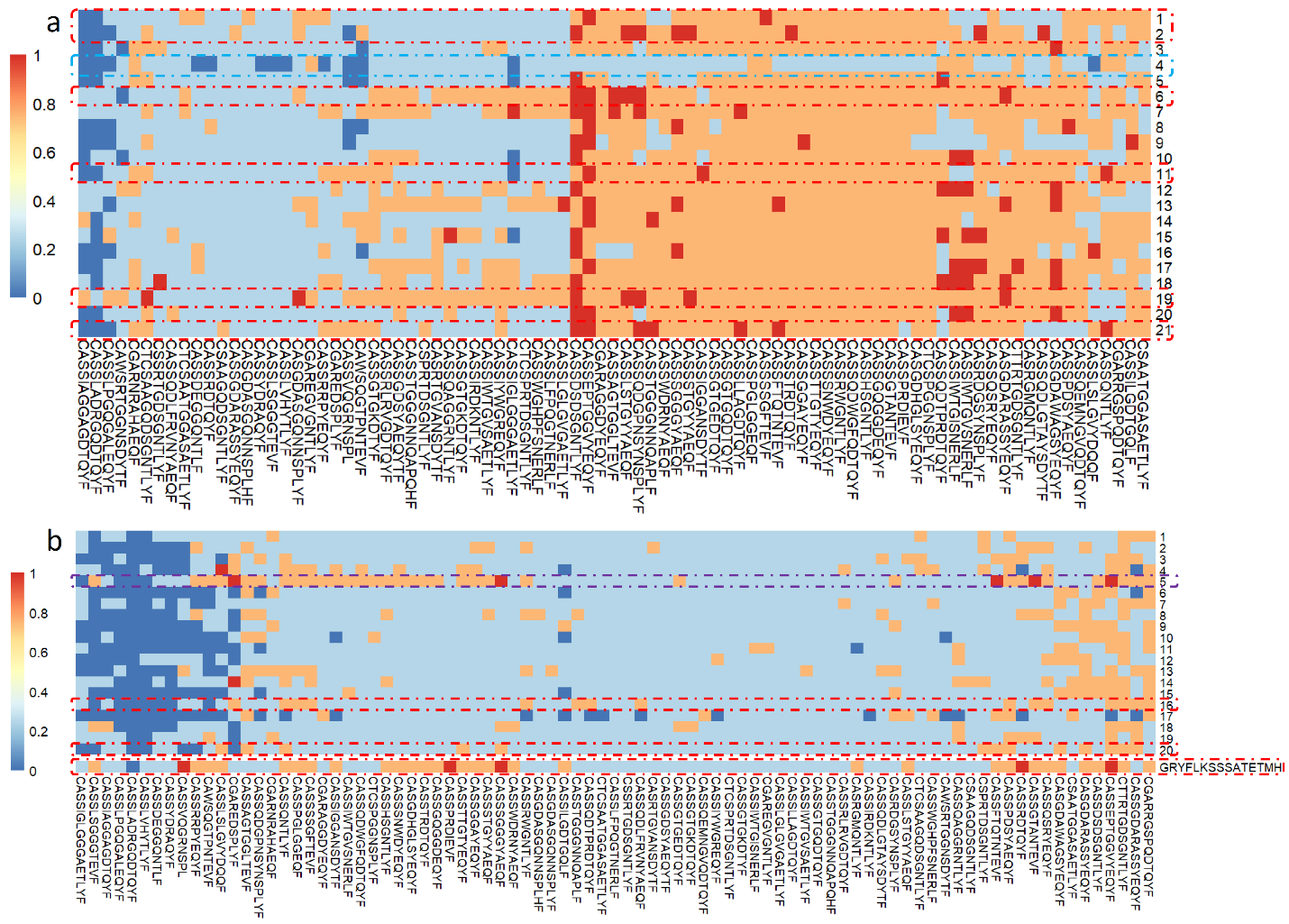
Figure. S2. Predicted T-cell binding ability. a The predicted T-cell interaction score for the top 21 MHC-I peptides screened by Neo-intline. Experimentally validated peptides were marked in the red dashed boxes, and MHC-I-4 was marked in the blue dashed boxes. b The predicted T-cell interaction score for 20 MHC-II peptides screened by Neo-intline. Experimentally validated peptides were marked in the red dashed boxes, and MHC-II-5 was marked in the purple dashed boxes.


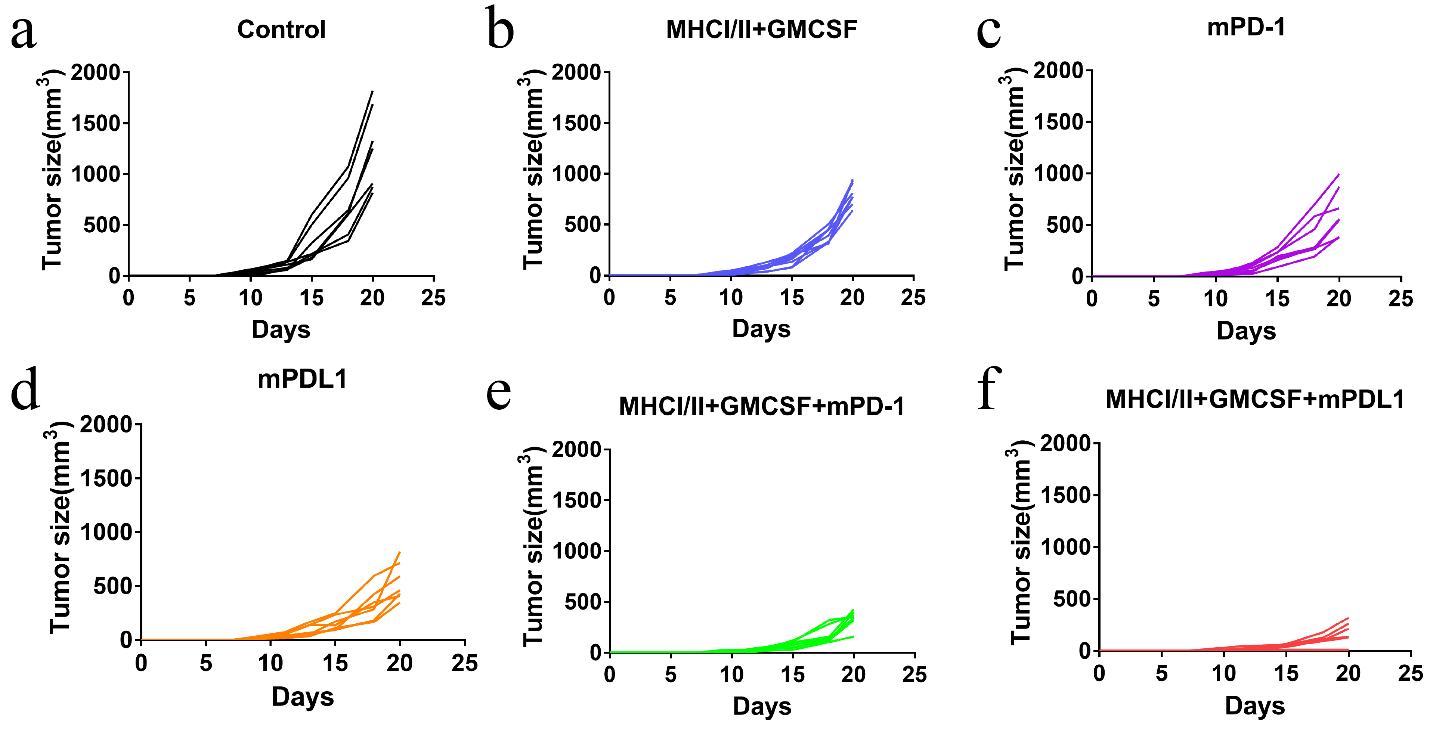
**Figure. S3. Tumor volume in Mice with different treatment groups. a** Control group. **b** The treatment group of MNA-I/II-10-G. **c** Treatment group of mPD-1. **d** The treatment group of mPDL1. **e** Treatment group of MNA-I/II-10-G with mPD-11 (MNA-I/II-10-G-mPD1). **f** Treatment group of MNA-I/II-10-G with mPDL1 (MNA-I/II-10-G-mPDL1).

**Table S1.** Ranking list of selected MHC-I peptides based on neoantigen probability score (NPS) with 45-mer length.

| Ranking | Final score | NeoantigenID | Gene | AAchange | MT.Peptide.Form | 45merPeptide |
| --- | --- | --- | --- | --- | --- | --- |
| 1 | 31.25757 | H2-Db_Acox3 | Acox3 | Lys18Met | SALWSDTPM | MGSLPEEKDSALWSDTPMGPLSAYRARASFNSGELLLFWD |
| 2 | 20.05251 | H2-Kb_Pcmtd1 | Pcmtd1 | Pro222Leu | VSFAPLVQL | RTGQNTWESKNILAVSFAPLVQLSKNDNGTPDSVGLPPCAVRNLQ |
| 3 | 3.186534 | H2-Ld_Hipk3 | Hipk3 | Ser702Phe | APAAATLTF | QQMLIPAWQQVTPMAPAAATLTFEGMAGSQRLGDWGKMIPHSNHY |
| 4 | 1.184672 | H2-Ld_Hmg20b | Hmg20b | Ala310Pro | LPRVASEHL | HGAIERDPAQHERLIARVKEILPRVASEHL |
| 5 | 0.898127 | H2-Ld_Rfc3 | Rfc3 | Arg213His | LPSTLAHRL | DICSVLSTVCRKEGLALPSTLAHRLAEKSCRNLRKALLMCEACRV |
| 6 | 0.538956 | H2-Db_Hsf2 | Hsf2 | Lys72Asn | YGFRNVVHI | PKYFKHNNMASFVRQLNMYGFRNVVHIESGIIKQERDGPVEFQHP |
| 7 | 0.484946 | H2-Kb_Pnp | Pnp | Asp248Glu | KVVMEYENL | IVARHCGLRVFGFSLITNKVVMEYENLEKANHMEVLDAGKAAAQT |
| 8 | 0.428271 | H2-Kd_Tmem39a | Tmem39a | Phe124Leu | YYLAALITV | PYNHPASCTSLNFHLIDYYLAALITVMLARRLVWALISEATKAGA |
| 9 | 0.358936 | H2-Db_Tmem39b | Tmem39b | Ala131Pro | FNLLMVTPI | SHPPSHTSLNFHLIDFNLLMVTPIVLGRRFIGSIVKEASQRGKVS |
| 10 | 0.247782 | H2-Kb_Xylb | Xylb | Leu388Met | GNMGFYFDV | ASCSWNKFSKALKSTAMGNNGNMGFYFDVMEITPEIIGRHRFNAE |
| 11 | 0.246384 | H2-Ld_Eef2 | Eef2 | Gly795Ala | LPVNESFAF | ESQVAGTPMFVVKAYLPVNESFAFTADLRSNTGGQAFPQCVFDHW |
| 12 | 0.18327 | H2-Ld_Klhl26 | Klhl26 | Glu487Ala | APMRAPRVL | DKKALQCYDPAADRWEPRAPMRAPRVLHAMLGAAGRIYALGGRMD |
| 13 | 0.156244 | H2-Kd_Heatr5a | Heatr5a | Ala1497Val | HYHDSWVLI | EGGAFYTAETSKSAKLHYHDSWVLILHAAALWLTSTGFADPDEGG |
| 14 | 0.068527 | H2-Kb_Wdr82 | Wdr82 | Ile221Leu | TNGSFIRLL | GLKFSNDGKLILISTNGSFIRLLDAFKGVVMHTFGGYANSKAVTL |
| 15 | 0.05485 | H2-Kb_Wdr5b | Wdr5b | Ala311Thr | KNIITSAAL | QRLQGHTDVVISAACHPTKNIITSAALENDKTIKVWSSDC |
| 16 | 0.044835 | H2-Kd_Wsb2 | Wsb2 | Pro390Gln | TYQVLALQI | SLKHLCRKALRSFLTTYQVLALQIPKKMKEFLTYRTF |
| 17 | 0.035381 | H2-Dd_Rad21 | Rad21 | Lys16Thr | RGPLATIWL | MFYAHFVLSKRGPLATIWLAAHWDKKLTKAHVFECNLE |
| 18 | 0.033909 | H2-Ld_Mllt4 | Mllt4 | Ser1126Asn | RPKNEGFEL | NQPSPMMQRISDRRGSGKPRPKNEGFELYNNSAQNGSPESPQMPW |
| 19 | 0.033237 | H2-Kb_Fat1 | Fat1 | Ile1940Met | IAMQNTTQL | YSITEGNIGEKFSMDHKTGTIAMQNTTQLRSRYELTVRASDGRFT |
| 20 | 0.032739 | H2-Kb_Shmt1 | Shmt1 | Phe119Val | ANVAVYTAL | AYHLDPQCWGVNVQPYSGSPANVAVYTALVEPHGRIMGLDLPDGG |
| 21 | 0.023378 | H2-Kb_Actn4 | Actn4 | Phe835Val | VTFQAFIDV | NRIMSVVDPNHSGLVTFQAFIDVMSRETTDTDTADQVIASFKVLA |
| 22 | 0.021458 | H2-Kb_Apbb2 | Apbb2 | Lys710Thr | CMLRYQTCL | WCEPNAANVSEAVQAACMLRYQTCLVARPPSQKVRPPPPPADSVT |
| 23 | 0.01538 | H2-Kd_Rfwd3 | Rfwd3 | Gly28Val | IYSHLQVPL | VGQASDLVPSEMDHEVIYSHLQVPLEGTIEPATPTEVVSNGAPLQ |
| 24 | 0.014557 | H2-Db_Chd6 | Chd6 | Asp1602Gly | YYIMNGPQL | DRDLLIGTAKHGLNRTDYYIMNGPQLSFLDAYRNYAQHKRTDTQA |
| 25 | 0.012402 | H2-Kb_Ap3d1 | Ap3d1 | Tyr418Cys | TNFEWCISI | LLTKIIDICSQSNYQHITNFEWCISILVELTRLEGTRHGHLIAAQ |
| 26 | 0.011639 | H2-Ld_Car11 | Car11 | Pro50Ser | VPGPSFWGL | APDPEDWWSYKENLQGNFVPGPSFWGLVNAAWSLCAVGKRQSPVD |
| 27 | 0.007785 | H2-Db_Braf | Braf | Cys264Arg | LMRVNYDQL | GFRCQTCGYKFHQRCSTEVPLMRVNYDQLDLLFVSKFFEHHPVPQ |
| 28 | 0.007474 | H2-Kd_Gamt | Gamt | Asp31Asn | AYDASNTHL | LFAPGEDCGPAWRAAPAAYDASNTHLQILGKPVMERWETPYMHAL |
| 29 | 0.002571 | H2-Kb_Kdm5b | Kdm5b | Pro516Ala | INYLHWGEA | SSFCWHIEDHWSYSINYLHWGEAKTWYGVPGYAAEQLENVMKKLA |
| 30 | 0.001143 | H2-Db_Ddb1 | Ddb1 | Leu438Ile | VLMINGEEV | DPGRETDDTLVLSFVGQTRVLMINGEEVEETELMGFVDDQQTFFC |
| 31 | 0.000577 | H2-Db_Dock9 | Dock9 | Val1877Met | FAYIQVTHM | QDSGKVNPKDLDSKFAYIQVTHMTPFFDEKELQERRTEFERCHNI |
| 32 | 0.000267 | H2-Ld_Atg7 | Atg7 | Phe287Ile | WPLRNILVL | KITVGVYDPCNLAQYPGWPLRNILVLAAHRWSGSFQSVEVLCFRD |

**Table S2.** Ranking list of selected MHC-II peptides based on neoantigen probability score (NPS) with 55-mer length.

| Rank-final | Final-score | NeoantigenID | Gene | AAchange | MT.Peptide.Form | 55merPeptide |
| --- | --- | --- | --- | --- | --- | --- |
| 1 | 12.42811037 | H2-IAd_Micall2_5 | Micall2 | Leu398Val | GVKTQLNVNSESSNT | VSPSARNTHLPGSQGQTASKGVKTQLNVNSESSNTAVTPAWTSSASKTQQAREKF |
| 2 | 8.246586042 | H2-IAd_Msantd3 | Msantd3 | Asp38Ala | VLECKKSAARTIALK | KYFSELEKSILLALVEKYKYVLECKKSAARTIALKQRTWQALAHEYNSQPSVSLR |
| 3 | 3.985213369 | H2-IAd_Dnmbp_5 | Dnmbp | Pro402Leu | KDLSTPDLSEEVNGV | GQDKDASGSSPDVDLERPLAKDLSTPDLSEEVNGVSSQPQVPIHPKVQKSQHYLT |
| 4 | 2.424910475 | H2-IAd_Szrd1_4 | Szrd1 | Arg99Leu | AEALRRILGSASPEE | VSSPNSTSRPALPVKSLAQREAEYAEALRRILGSASPEEEQEKPILDRPTRISQP |
| 5 | 1.998638743 | H2-IAd_Heatr5a_3 | Heatr5a | Ala1497Val | HDSWVLILHAAALWL | SQLPTEGGAFYTAETSKSAKLHYHDSWVLILHAAALWLTSTGFADPDEGGANLSR |
| 6 | 1.785484809 | H2-IAb_Dennd4a_2 | Dennd4a | Ser1512Ala | PGRYFLKSSSATETM | GNLFLPFLNVEIRDLRRPGRYFLKSSSATETMHFASRTRQSCISASASGLDTSSL |
| 7 | 1.55396958 | H2-IAd_Fam207a_2 | Fam207a | Ser168Ile | RPVELIRMTTVQRQQ | ELQELEAGRQRQQARRRVTSKPRPVELIRMTTVQRQQLLEEERTRFQKLLASPTY |
| 8 | 1.386427836 | H2-IAd_1700056E22Rik_5 | 1700056E22Rik | Pro182Thr | PITRTLSIRGVTTGR | KEPELGLNSQNERRLRSMEGPTPALPITRTLSIRGVTTGRGMSPVKRSGKLSKPA |
| 9 | 1.318832226 | H2-IAd_Atp6v1h_10 | Atp6v1h | Lys147Thr | ARIIATLAAWGKELM | STAWPYFLPMLNRQDPFTVHMAARIIATLAAWGKELMEGSDLNYYFNWIKTQLSS |
| 10 | 1.014234919 | H2-IAd_Spire1_9 | Spire1 | Arg554Leu | PLLSIARFSTKSRSV | SLGPSALQRGESCSRSEKPSTSHHRPLLSIARFSTKSRSVDKSDEELQFPKELME |
| 11 | 0.740452065 | H2-IAd_Fkrp_5 | Fkrp | Gly273Arg | SRRAALLRSLRIRLV | PPLATAHARWKAEREGRSRRAALLRSLRIRLVSWEGGRLEWFGCSKESARCFGTV |
| 12 | 0.567198244 | H2-IAd_Ogdh | Ogdh | Asp993Ala | DYVKPRLRTTIARAK | AELAWCQEEHKNQGYYDYVKPRLRTTIARAKPVWYAGRDPAAAPATGNKKTHLTE |
| 13 | 0.404834935 | H2-IAd_Apbb1_3 | Apbb1 | Cys654Tyr | NAASLSEAVQAAYML | AGPASFCCHMFWCEPNAASLSEAVQAAYMLRYQKCLDARSQTSTSCLPAPPAESV |
| 14 | 0.327582106 | H2-IAd_Wdr5b_2 | Wdr5b | Ala311Thr | CHPTKNIITSAALEN | TKEIVQRLQGHTDVVISAACHPTKNIITSAALENDKTIKVWSSDC |
| 15 | 0.287398186 | H2-IAd_Rassf8_2 | Rassf8 | Gln178Arg | LKRLIRLQTGKLRAI | FRQKVLSNCRATAEELKRLIRLQTGKLRAIEKQLESSEAEIRFWEQKYSCSLEEE |
| 16 | 0.25409264 | H2-IAb_Commd4_3 | Commd4 | Ser73Gly | VLGFILSSAAKHSVD | IDYEKILKLTADAKFESGDVKATVAVLGFILSSAAKHSVDSDSLSSELQQLGLPK |
| 17 | 0.252801158 | H2-IAb_Wdr53 | Wdr53 | Ser154Thr | SSVAFRPQRPQSLVT | KVTRSLKRHSNICSSVAFRPQRPQSLVTCGLDMQVMLWSLQKARPVWITNLQEDE |
| 18 | 0.236513174 | H2-IAd_Golgb1_3 | Golgb1 | Glu2855Asp | DVQSLKKAMSSLQND | WSELEKFRKSEEGKQRAAAPSAASSPADVQSLKKAMSSLQNDRDRLLKELKNLQQ |
| 19 | 0.234667321 | H2-IAb_Rab3gap1_3 | Rab3gap1 | Asp539Tyr | TSLSYSTTSAYPGDA | LHQKLQMLNCCIERKKARDEGKKTSLSYSTTSAYPGDAGKTGGQLGLDHLRDTEK |
| 20 | 0.216452388 | H2-IAb_Chd6_5 | Chd6 | Asp1602Gly | DYYIMNGPQLSFLDA | ECGKHDRDLLIGTAKHGLNRTDYYIMNGPQLSFLDAYRNYAQHKRTDTQAPGSLC |
| 21 | 0.141945872 | H2-IAd_Med14_3 | Med14 | Leu1451Arg | TIFAAVRDLMANRTL | LKRFAEMNPPRQGECTIFAAVRDLMANRTLPPGGRP |
| 22 | 0.139586896 | H2-IAd_Vps13c_6 | Vps13c | Ser1256Pro | VIVIPQSSLPTNAVV | DLAQRSFRVSVDIDLKAPVIVIPQSSLPTNAVVVDLGLIRVHNRFSLVSGEDTAN |
| 23 | 0.131959993 | H2-IAd_Fbxl14_3 | Fbxl14 | Gly186Ala | AIGHLAGMTRSAAEG | TGLLLIAWGLQRLKSLNLRSCRHLSDVAIGHLAGMTRSAAEGCLGLEQLTLQDCQ |
| 24 | 0.127654403 | H2-IAd_Ap5b1_6 | Ap5b1 | Lys624Thr | LLSHLSSSTLGMALG | LARQLENADGRDHARLYYVLLSHLSSSTLGMALGPSLAAPALASSLMAENQGFSS |
| 25 | 0.112296279 | H2-IAd_Rab3gap2 | Rab3gap2 | Leu1193Phe | AFFKDFTSIQLLPSG | ASMRFSLKSVKPLALFDSKGKNAFFKDFTSIQLLPSGEMDPNFISVRQQFLLKVV |
| 26 | 0.098754094 | H2-IAb_Ppp2r1b_3 | Ppp2r1b | Glu309Asp | EADVRAAAAHKVREL | LQKAVGPKIALSDLIPAFQSLLRDCEADVRAAAAHKVRELCENLPAEGRETVIMN |
| 27 | 0.094885316 | H2-IAd_Dlg4_3 | Dlg4 | Arg126Gln | VQEVTHSAAVEALKE | IIPGGAAAQDGRLRVNDSILFVNEVDVQEVTHSAAVEALKEAGSIVRLYVMRRKP |
| 28 | 0.08183301 | H2-IAd_Tm9sf3_10 | Tm9sf3 | Tyr382His | HHASRAIPFGTMVAV | KQMFIGAFLIPAMVCGTAFFINFIAIYHHASRAIPFGTMVAVCCICFFVILPLNL |
| 29 | 0.080326883 | H2-IAd_Fzd7_4 | Fzd7 | Gly304Ala | YFMVAVAHVAAFLLE | MRRFSYPERPIIFLSGCYFMVAVAHVAAFLLEDRAVCVERFSDDGYRTVAQGTKK |
| 30 | 0.073227831 | H2-IAd_Orc2_5 | Orc2 | Phe278Val | NLLRKVVPSFSAEIE | VLTSDRTLQRLRRARVDQKTLHNLLRKVVPSFSAEIERLNQQHEKLFHKWMLQLH |
| 31 | 0.066024823 | H2-IAb_Gpatch4_2 | Gpatch4 | Gly192Ala | FLAQLKGSKALATSQ | ITMKAKLARLEAQEQAFLAQLKGSKALATSQPLTDSEPSQKKKKKRKQKEGEEAA |
| 32 | 0.058061647 | H2-IAd_Myh9 | Myh9 | Leu1089Val | AKKEEELQAAVARVE | DQIAELQAQIAELKMQLAKKEEELQAAVARVEEEAAQKNMALKKIRELETQISEL |
| 33 | 0.05601454 | H2-IAd_Ncapg_3 | Ncapg | Leu431Arg | ILQETRILPTTPISL | QLILIMKSLDTSEEGGRKRLLAILQETRILPTTPISLVSLIVERLLHIIRDDNER |
| 34 | 0.055485876 | H2-IAb_Cpsf3l | Cpsf3l | Asp314Asn | FDRTFANNPGPMVVF | NQKIRKTFVQRNMFEFKHIKAFDRTFANNPGPMVVFATPGMLHAGQSLQIFRKWA |
| 35 | 0.055027954 | H2-IAd_Arhgap18_2 | Arhgap18 | Gln147Leu | FLSTLTRTQAAAVLK | LFGESIDDPQESILFLSTLTRTQAAAVLKRVETVSQTLRKKNKQHHIRDVRDIFA |
| 36 | 0.05428505 | H2-IAd_Ubr3 | Ubr3 | Thr322Ala | AAGASTLAAQGFAGA | YLIALKSSGLTYPEDKLVYGVQEPAAGASTLAAQGFAGATGTLGQIDSSDEEDQD |
| 37 | 0.054006917 | H2-IAd_Acox3_6 | Acox3 | Lys18Met | MGPLSAYRARASFNS | MGSLPEEKDSALWSDTPMGPLSAYRARASFNSGELLLFWDGQDVI |
| 38 | 0.052885082 | H2-IAb_Rpl12_4 | Rpl12 | Ile82Asn | IEVVPSASALINKAL | GLRITVKLTIQNRQAQIEVVPSASALINKALKEPPRDRKKQKNIKHSGNITFDEI |
| 39 | 0.050205793 | H2-IAd_Dennd5a_3 | Dennd5a | Asp1250Ala | ITAHMYEAVALIKDH | CLGARDHLLHHWIALLADCPITAHMYEAVALIKDHTLVNSLIRVLQTLQEFNITL |
| 40 | 0.048888394 | H2-IAb_Ddx23_5 | Ddx23 | Val602Ala | TAMFTATMPPAVERL | KPDTDEAEDPEKMLANFESGKHKYRQTAMFTATMPPAVERLARSYLRRPAVVYIG |
| 41 | 0.045959991 | H2-IAd_Car11 | Car11 | Pro50Ser | SFWGLVNAAWSLCAV | AHIGPAPDPEDWWSYKENLQGNFVPGPSFWGLVNAAWSLCAVGKRQSPVDVELKR |
| 42 | 0.045569116 | H2-IAd_Hjurp_7 | Hjurp | Pro315His | MSRLLRSKLSCIIST | LHKNRTHCPRSKPSQRSARKGPASCSEHGKEAGILRDYGNLLHVAPHKTGLELKS |
| 43 | 0.040058991 | H2-IAd_Slc25a1_4 | Slc25a1 | Gly130Ala | LLCGLAAGVAEAVVV | GMFEFLSNHMRDAQGRLDSRRGLLCGLAAGVAEAVVVVCPMETIKVKFIHDQTSS |
| 44 | 0.039150735 | H2-IAd_Ctsd | Ctsd | Gly403Ser | DRDNNRVSFANAVVL | PSGPLWILGDVFIGSYYTVFDRDNNRVSFANAVVL |
| 45 | 0.037774736 | H2-IAd_Ttc28_4 | Ttc28 | Gly2374Ala | VLSLLNLSPRHAKEE | RKTCRGAPGTLTSKRDVLSLLNLSPRHAKEEGGADRLELKELSVQRHDEVPPKVP |
| 46 | 0.03597131 | H2-IAd_Lyst | Lyst | Gln3359His | YKQKGKASVHAINVF | ESDHVSQNICHWIDLVFGYKQKGKASVHAINVFHPATYFGMDVSAVEDPVQRRAL |
| 47 | 0.032648042 | H2-IAd_Tctn3_5 | Tctn3 | Phe332Val | FGIQKVSVSVRQTNL | TCQNIVSQVVYEIETNGTFGIQKVSVSVRQTNLTVKPGVSLQQDFIVHFRAFQQR |
| 48 | 0.032486971 | H2-IAd_Pnrc1 | Pnrc1 | Thr196Ile | KSKYNLPLTKIISAK | LVHGIHLCEQPKINRQKSKYNLPLTKIISAKRNESDFWQDSASSDRMQKQEKKSF |
| 49 | 0.031021538 | H2-IAd_Btaf1 | Btaf1 | Val863Asp | QVLQLRVHTFAACAD | QVQMTVAETNQEWQVLQLRVHTFAACADVSLQQLPEKLNPIIKPLMETIKKEENT |
| 50 | 0.029626836 | H2-IAd_B3galt6_3 | B3galt6 | Arg228Leu | LRLSLEYLRAWHSED | LCDYYLPYALGGGYVLSADLVHYLRLSLEYLRAWHSEDVSLGTWLAPVDVQREHD |
| 51 | 0.028462708 | H2-IAd_Cnst_3 | Cnst | Gly59Ala | LTTSEGAMARATVSE | LPSVSDENENQLAGDGPAGLTTSEGAMARATVSEQDSLNNNESFPSSCEAAPTEN |
| 52 | 0.023204089 | H2-IAd_Map1s | Map1s | Phe881Val | HLDQNVFLRVRALCY | APLAAGSPVYLDLAYLPGGGAGHLDQNVFLRVRALCYVISGQGQRQEEGLRAVLD |
| 53 | 0.022626932 | H2-IAb_Ermp1 | Ermp1 | Ser792Arg | DSIKLTFEATGPRHM | NPAHFRLVSKEKMPWDSIKLTFEATGPRHMSFYVRTHKGSTLSQWSLGNGIPVTS |
| 54 | 0.021126695 | H2-IAd_BC068281_3 | BC068281 | Lys227Thr | LPLHTLCSLNASEAL | FPVNCSIRSIEATGNSQVAIATELPLHTLCSLNASEALDGPPNGDDGSVHTRPVD |
| 55 | 0.020943537 | H2-IAd_Tnpo1_3 | Tnpo1 | Gly425Ala | VVKESAILVLGAIAE | YRDELLPHILPLLKELLFHHEWVVKESAILVLGAIAEGCMQGMIPYLPELIPHLI |
| 56 | 0.020551011 | H2-IAd_Hmg20b | Hmg20b | Ala310Pro | RLIARVKEILPRVAS | YMARLHGAIERDPAQHERLIARVKEILPRVASEHL |
| 57 | 0.020304532 | H2-IAd_Zfat | Zfat | Ala195Val | LSGVKKPIISVVLTA | KASKRPRAQKTEKVQKISGKEAGQLSGVKKPIISVVLTAHEAIPGATKIIPVEAG |
| 58 | 0.019864453 | H2-IAd_Wiz_4 | Wiz | Lys467Met | TARMMFSGLATPSLP | PLTKKLPPPPGSPLGHSPTASPPPTARMMFSGLATPSLPKKLKPEHMRVEIKREM |
| 59 | 0.019165661 | H2-IAd_Hipk3 | Hipk3 | Ser702Phe | QQVTPMAPAAATLTF | WSGRTQQMLIPAWQQVTPMAPAAATLTFEGMAGSQRLGDWGKMIPHSNHYNSVMP |
| 60 | 0.018793683 | H2-IAd_Nacc1_5 | Nacc1 | Pro220Ala | ALNRMPQALSMATAT | GGSGGNNGSRKMAKFSTPDLALNRMPQALSMATATAAVAVVAVGGCVSGPSMSER |
| 61 | 0.01863565 | H2-IAd_Pcmtd1_3 | Pcmtd1 | Pro222Leu | NILAVSFAPLVQLSK | LTQIMRTGQNTWESKNILAVSFAPLVQLSKNDNGTPDSVGLPPCAVRNLQDLARI |
| 62 | 0.018557403 | H2-IAd_Eif3m_9 | Eif3m | Leu127Arg | RIKVAASCGAIQYIP | SLRLQLLSNLFHGMDKNTPVRYTVYCSRIKVAASCGAIQYIPTELDQVRKWISDW |
| 63 | 0.018322786 | H2-IAd_Dock10 | Dock10 | Lys1601Arg | RSIVRSHLQLIKAVS | HRSRLTQMEASALLYFFMRKNFEFNKQRSIVRSHLQLIKAVSQLIADAGIGGSRF |
| 64 | 0.018237003 | H2-IAd_Ddx5 | Ddx5 | Asn562Thr | NFVSAGIQTSFRTGT | YSAANYTNGSFGSNFVSAGIQTSFRTGTPTGTYQNGYDSTQQYGSNVANMHNGMN |
| 65 | 0.016969747 | H2-IAd_Numa1 | Numa1 | Lys1331Thr | ETFFQKEQALSALQL | SENSRQELASQAERAEELGQELKAWQETFFQKEQALSALQLEHTSTQALVSELLP |
| 66 | 0.016434499 | H2-IAb_Jmy_2 | Jmy | Ser860Cys | PKSASAPAAHLFDCS | EKDALRTEGNERSIPKSASAPAAHLFDCSQLVSARKKLRKTVEGLQRRRVSSPMD |
| 67 | 0.015139591 | H2-IAd_Mfhas1 | Mfhas1 | Pro622Ala | YLLNHRLQILSPVLA | SDKNLRRRKAHFQYLLNHRLQILSPVLAVSCRDPLQLQRLRDKLLSVAEHREIFP |
| 68 | 0.013346675 | H2-IAd_Herc2_4 | Herc2 | Cys4450Phe | VFGQMFAKMSSFSPD | NRIQVKRSRSKGGLAGPDGTKSVFGQMFAKMSSFSPDSLLLPHRVWKVKFVGESV |
| 69 | 0.010769874 | H2-IAd_Klhl26 | Klhl26 | Glu487Ala | RWEPRAPMRAPRVLH | GVSAEDKKALQCYDPAADRWEPRAPMRAPRVLHAMLGAAGRIYALGGRMDHVDRC |
| 70 | 0.009614249 | H2-IAd_Sema3b_3 | Sema3b | Leu663Val | QPLRRLVLHVVSAAQ | RQDSGVYLCVAVEQGFSQPLRRLVLHVVSAAQAERLARAEEAAAPAPPGPKLWYR |
| 71 | 0.008700432 | H2-IAd_Rabl2_6 | Rabl2 | Ala180Pro | AIRLAVPYKESSQDF | SLPLYFVSAADGTNVVKLFNDAIRLAVPYKESSQDFMDEVLQELENFKLEQKEED |
| 72 | 0.0084973 | H2-IAd_Pdpr_6 | Pdpr | Thr669Met | ANGIRVMSMMHTGEP | MTPDHFPTLFCKEMSVGYANGIRVMSMMHTGEPGFMLYIPIEYALHVYNEVMSVG |
| 73 | 0.008030758 | H2-IAd_Rnmt | Rnmt | Ser136Leu | QKLEEGHSLAVAAHY | LNQTKRKLQPQDDEVPQKLQKLEEGHSLAVAAHYNELQEVGLAKRSQSRIFYLRN |
| 74 | 0.007431111 | H2-IAd_Tmem39a | Tmem39a | Phe124Leu | DYYLAALITVMLARR | TVWWYPYNHPASCTSLNFHLIDYYLAALITVMLARRLVWALISEATKAGAASTVH |
| 75 | 0.007062546 | H2-IAd_Mapk13_5 | Mapk13 | Glu245Gly | ILKVTGVPGAGFVQK | TGKTLFKGKDYLDQLTQILKVTGVPGAGFVQKLKDKAAKSYIQSLPQSPKKDFTQ |
| 76 | 0.006596553 | H2-IAd_C77080_4 | C77080 | Ala903Thr | TPSSGLHAAVRLKAS | IPNPSPGSSAPQKPLRRALSGRASPVTTPSSGLHAAVRLKASSLAASESPASALP |
| 77 | 0.006546219 | H2-IAd_Wwc1_2 | Wwc1 | Asn317Thr | EAKRRIATLKIQLAK | FSTSSNNQLAEKVRLRLRYEEAKRRIATLKIQLAKLDSEAWPGVLDSERDRLILI |
| 78 | 0.004771933 | H2-IAd_Atg7 | Atg7 | Phe287Ile | QYPGWPLRNILVLAA | QGQRTKITVGVYDPCNLAQYPGWPLRNILVLAAHRWSGSFQSVEVLCFRDRTMQG |
| 79 | 0.004487778 | H2-IAd_Synm | Synm | Leu285Val | LEDEKEALTVAMADR | MREEYGMQAEERQRVIDSLEDEKEALTVAMADRLRDYQELLQVKTGLSLEVATYR |
| 80 | 0.004416218 | H2-IAd_Wipi2 | Wipi2 | Thr304Ala | QVTEMFNQGRAFAAV | FGKVLMASTSYLPSQVTEMFNQGRAFAAVRLPFCGHKNICSLTTIQKIPRLLVGA |
| 81 | 0.003442697 | H2-IAd_Pds5a_6 | Pds5a | Ile1204Met | PVRIMSVTPVKNIDT | AGSRSREQSSEASETGVSENEENPVRIMSVTPVKNIDTVKNKEINSDQSTQGNIS |
| 82 | 0.00342571 | H2-IAd_Parp14_7 | Parp14 | Asp1360His | VVAKAIIHAIEEFVQ | NYSSICLPAIGTGNAQQDPNVVAKAIIHAIEEFVQKKSVQAVKRVKVVIFQPHIL |
| 83 | 0.003119121 | H2-IAd_Zmiz2_2 | Zmiz2 | His447Leu | EPFRLQHNLAVSNLV | ELRLTFPVRDGVVLEPFRLQHNLAVSNLVFQLRDSVYKTLMLRPDLELQFKCYHH |
| 84 | 0.002637365 | H2-IAd_Haus6_3 | Haus6 | Leu176Val | CLARSHVARNRFVQI | HFAETFNVKPQDMHKCLARSHVARNRFVQILQREHYVMQKYQENVNLSVKQVRNA |
| 85 | 0.002523375 | H2-IAd_Wdr82 | Wdr82 | Ile221Leu | KLILISTNGSFIRLL | TCEWTGLKFSNDGKLILISTNGSFIRLLDAFKGVVMHTFGGYANSKAVTLEASFT |
| 86 | 0.002483702 | H2-IAd_Lmbr1 | Lmbr1 | Ala137Ser | SGLKKGIRARILETL | NLASLFSNLCLFVLMPFAFFFLESEGFSGLKKGIRARILETLVMLLLLALLILGM |
| 87 | 0.002470279 | H2-IAd_Mcm6 | Mcm6 | Asn454Thr | HEFVIEAGALMLADT | AGLTAAVVRDEESHEFVIEAGALMLADTGVCCIDEFDKMDMRDQVAIHEAMEQQT |
| 88 | 0.002443934 | H2-IAb_Ddit4l_2 | Ddit4l | Gly163Ala | DFFFSRGRFSSALKR | LTLVFKQESCPWTSLKDFFFSRGRFSSALKRTLILSSGFRLVKKKLYSLIGTTVI |
| 89 | 0.001824226 | H2-IAd_Serac1_4 | Serac1 | Pro533Ser | GIIFYSVSHHGSRLA | KMLLEASKKPELNALINNTRGIIFYSVSHHGSRLAEYSVNIRYLLFPSLEVKELS |
| 90 | 0.001786999 | H2-IAd_Pbk_9 | Pbk | Val145Asp | DALHMARGLKYLHQE | GEKSLNDLIEERNKDSGSPFPAAVILRDALHMARGLKYLHQEKKLLHGDIKSSNV |
| 91 | 0.001308974 | H2-IAd_Pelp1_6 | Pelp1 | Leu402Pro | LRFGALISRPLPQVL | LEALDLLSALILACGSRLLRFGALISRPLPQVLNAWSTGRDTLAPGQERPYSTIR |
| 92 | 0.001119309 | H2-IAb_Dmxl1_2 | Dmxl1 | Asn2546Ser | SEESVSAGPAILRHK | WEQVLLRRLEIHGGPPQNYISSHTSEESVSAGPAILRHKALLEPTNTPFKSKNHL |
| 93 | 0.001035211 | H2-IAd_Hipk1_5 | Hipk1 | Glu413Gly | YISQTQGLPAGYLLS | FLGWPLYPGASEYDQIRYISQTQGLPAGYLLSAGTKTTRFFNRDPNLGYPLWRLK |
| 94 | 0.001000051 | H2-IAd_Mrpl2 | Mrpl2 | Lys62Arg | YRPVHMSADRSARFV | SNVLLQLPPALVSPSYRPVHMSADRSARFVSWKSRTKYTVKPVKMRKSGGRDHTG |
| 95 | 0.000968029 | H2-IAd_Nsun2_2 | Nsun2 | Lys138Met | NLSRKILRMSPLLAK | KVEVPQPLSWYPEELAWHTNLSRKILRMSPLLAKFHQFLVSETESGNISRQEAVS |
| 96 | 0.000903256 | H2-IAd_Cdca7l_4 | Cdca7l | Met207Val | VNIKENKAMLAQLLA | KEDSASDAEDESRAESQENSDALLKRAVNIKENKAMLAQLLAELNSVPDFFPVRT |
| 97 | 0.000900998 | H2-IAd_Gpaa1 | Gpaa1 | Glu142Gly | NVYGILRAPRSASTG | PDETHERYMVSGTNVYGILRAPRSASTGSLVLTVPCGPDATNSQAVGLLLALAAH |
| 98 | 0.000814011 | H2-IAd_Piga_4 | Piga | Lys88Asn | NVYYLPLRVMYNQST | IERGHKVITVTHAYGNRKGVRYLTNGLNVYYLPLRVMYNQSTATTLFHSLPLLRY |
| 99 | 0.000812095 | H2-IAd_Rpl13a_6 | Rpl13a | Ala24Gly | LLGRLAAIVGKQVLL | MAEGQVLVLDGRGHLLGRLAAIVGKQVLLGRKVVVVRCEGINISGNFYRNK |
| 100 | 0.000793121 | H2-IAd_Xpc_2 | Xpc | Leu600Val | WWAETVRPYRSLLTE | DVTQRYDPAWMTATRKCRVDAEWWAETVRPYRSLLTEREKKEDQEFQAKHLDQPL |
| 101 | 0.000627063 | H2-IAd_Tyro3_3 | Tyro3 | Ile381Ser | KDLSLRVCASNAIGD | QENGTQDELMVEGTRANLTDWDPQKDLSLRVCASNAIGDGPWSQPLVVSSHDHAG |
| 102 | 0.000561587 | H2-IAd_9030624J02Rik_2 | 9030624J02Rik | Glu549Lys | KRKVNTVLADVIKHM | TKLKSPQDYINCAEVWVEYTCRHFTKRKVNTVLADVIKHMTPDRAFEDSYPQLQS |
| 103 | 0.000496586 | H2-IAb_Sec16a_5 | Sec16a | Ser970Phe | PFEFVSSPAGNTSVM | SQSASWLVQQLSPQTPQSPHPNAEKGPFEFVSSPAGNTSVMLVPPASSTLVPNSN |
| 104 | 0.00041218 | H2-IAd_Mkrn1_2 | Mkrn1 | Asn346Tyr | ECRITSNFVIPSEYW | SAKQFESKIIKSCPECRITSNFVIPSEYWVEEKEEKQKLIQKYKEAMSNKACRYF |
| 105 | 0.000409746 | H2-IAd_Nbea_3 | Nbea | Thr2251Ile | KVVYSLPRVGVGISY | RTSVMFNFPDQATVKKVVYSLPRVGVGISYGLPQARRISLATPRQLYKSSNMTQR |
| 106 | 0.000381834 | H2-IAd_Gamt | Gamt | Asp31Asn | GPAWRAAPAAYDASN | SAASPLFAPGEDCGPAWRAAPAAYDASNTHLQILGKPVMERWETPYMHALAAAAA |
| 107 | 0.000330186 | H2-IAd_Def8 | Def8 | Arg279Gly | HCHWNDLAVIPAGVV | SEARQCDYTGQYYCSHCHWNDLAVIPAGVVHNWDFEPRKVSRCSMRYLALMVSRP |
| 108 | 0.000328412 | H2-IAb_Srd5a3_3 | Srd5a3 | Phe157Leu | LECFYVSVFSNAAIH | SRMPAAELALSAFLVLVFLWVHSLRRLLECFYVSVFSNAAIHVVQYCFGLVYYVL |
| 109 | 0.00032762 | H2-IAb_Oaz1_2 | Oaz1 | Arg24Gly | GSATLHASRTMPLLS | MVKSSLQRILNSHCFAREKEGDKGSATLHASRTMPLLSQHSRGGCSSESSR |
| 110 | 0.000321484 | H2-IAd_Abca8b | Abca8b | Met250Lys | TYYVSINVARERKRK | FFIFTCIISFSPITYYVSINVARERKRKKGLMMMMGLRDPAFWLSWGLLYAGFVF |
| 111 | 0.000311607 | H2-IAd_Jmjd1c | Jmjd1c | Leu1895Pro | HDHKHLMPTQIIPGS | RKSSRDKELYAWMKCVKGQPHDHKHLMPTQIIPGSVLTDLLDAMHILREKYGIKS |
| 112 | 0.000299515 | H2-IAd_Shmt1 | Shmt1 | Phe119Val | NVAVYTALVEPHGRI | KRALQAYHLDPQCWGVNVQPYSGSPANVAVYTALVEPHGRIMGLDLPDGGHLTHG |
| 113 | 0.00029489 | H2-IAb_Fam193a_4 | Fam193a | Glu245Asp | CIYRQAGTPLADDQD | PQQLQNYWSEVRYMVRCIYRQAGTPLADDQDQSLVPDKEGVKELVDRLCERDPYQ |
| 114 | 0.000258794 | H2-IAd_Ngrn_7 | Ngrn | Ala162Pro | KPLSAGRSVSGLLMA | RQDQKVLKKAGFTREIGQLPVSEDTLKPLSAGRSVSGLLMAGDEVSSKSQNHSTA |
| 115 | 0.000200057 | H2-IAb_Ehbp1l1_4 | Ehbp1l1 | Pro1343Ala | TYRVGNAQPSLADCL | GQELQLVQLEGGGGSGTYRVGNAQPSLADCLDAGDLAQRLREHGAEVPTEPKEAV |
| 116 | 0.000172429 | H2-IAd_Tubd1_2 | Tubd1 | Lys211Met | NDAVHMICAKRMNIK | NYNSILTLSHLYRSSDALLIHENDAVHMICAKRMNIKQISFRDLNQVLAHQLGSV |
| 117 | 0.000156581 | H2-IAd_Ipo13 | Ipo13 | Glu441Gly | YVYEMLGAGLLSNLY | SDEKEQFRIYRVDISDTLMYVYEMLGAGLLSNLYDKLGRLLTSSEEPYSWQHTEA |
| 118 | 0.00013409 | H2-IAb_Kdm5b | Kdm5b | Pro516Ala | AKTWYGVPGYAAEQL | VGMCFSSFCWHIEDHWSYSINYLHWGEAKTWYGVPGYAAEQLENVMKKLAPELFV |
| 119 | 0.000129108 | H2-IAb_Nolc1 | Nolc1 | Lys495Asn | PAKNKAAGGAVSTPA | QAAGDSSSDSDSSSSEEEEKTPKPPAKNKAAGGAVSTPAPGKKAEAKSSSSSSSS |
| 120 | 0.00011915 | H2-IAd_Dock9_2 | Dock9 | Val1877Met | LDSKFAYIQVTHMTP | NVKMIQDSGKVNPKDLDSKFAYIQVTHMTPFFDEKELQERRTEFERCHNIRRFMF |
| 121 | 9.27E-05 | H2-IAb_Lrp10_5 | Lrp10 | Thr190Pro | NLNPAPAPPLACNLT | IDACGDGSDEAGCSSDPFPNLNPAPAPPLACNLTLEDFYGVFSSPGYSHLASVSH |
| 122 | 6.23E-05 | H2-IAd_Eef2_2 | Eef2 | Gly795Ala | FVVKAYLPVNESFAF | GHVFEESQVAGTPMFVVKAYLPVNESFAFTADLRSNTGGQAFPQCVFDHWQILPG |
| 123 | 3.66E-05 | H2-IAd_Lrrc8a_4 | Lrrc8a | Arg545Cys | CLKVLRLKSNLSKLP | LEELHLTGNLSAENNRYIVIDGLRELKCLKVLRLKSNLSKLPQVVTDVGVHLQKL |
| 124 | 3.18E-05 | H2-IAd_Scaf8 | Scaf8 | Leu116Val | DKSKIVRVLNVWQKN | SNNIISTFQNLYRCPGDDKSKIVRVLNVWQKNNVFKSEIIQPLLDMAAGIPPPVV |
| 125 | 2.61E-05 | H2-IAd_Klhl22_5 | Klhl22 | Phe179Val | YILKNVVAFSRTDKY | ILDVYRLADLFDLNHLTQQLDTYILKNVVAFSRTDKYRQLPLEKVYSLLSSNRLE |
| 126 | 2.58E-05 | H2-IAd_Acot8 | Acot8 | Gln133Arg | ASFQQMRPSPLQHQF | ASFSVRAVKAVQHGKAIFICQASFQQMRPSPLQHQFSMPSVPPPEDLLDHEALID |
| 127 | 2.02E-05 | H2-IAd_Nfkbiz_6 | Nfkbiz | Ala500Gly | AFQVAVAGNQHLIVQ | LARKMNALHMLDIKEHNGQSAFQVAVAGNQHLIVQDLVNLGAQVNTTDCWGRTPL |
| 128 | 1.70E-05 | H2-IAd_Supt3_3 | Supt3 | Lys353Arg | HRIGPLSPFTSAYRR | TAACGVEAHSDAIQPCHIREAIRRYGHRIGPLSPFTSAYRRSGMAFLAC |
| 129 | 1.67E-05 | H2-IAd_Tmem246_4 | Tmem246 | Leu11Pro | STSPAAMPLRRLRRL | MTTSTSPAAMPLRRLRRLSWGSTAVQLFILTVVTFGLL |
| 130 | 1.51E-05 | H2-IAd_Rint1 | Rint1 | Asn174Asp | DIQQYLMTNSVPEAA | AMITQMEEIERHLAYLKWVSQTEELSDDIQQYLMTNSVPEAASLLVTMTELDIQL |
| 131 | 7.70E-06 | H2-IAd_Ddx19b_4 | Ddx19b | Lys176Thr | LAVLSQVEPANTFAQ | LIAQSQSGTGKTAAFVLAVLSQVEPANTFAQCLCLSPTYELALQTGKVIEQMGKF |
| 132 | 6.50E-06 | H2-IAd_Mrpl1 | Mrpl1 | Met267Arg | ERQNFLSTKIATLDR | ELFKTAHEIMVDEERQNFLSTKIATLDRPSDQIAANLQAVINEVCKHRPLNLGPF |
| 133 | 6.10E-06 | H2-IAb_Eml5_2 | Eml5 | Ala1005Ser | LSTHPYLPICATVSD | GEILEVDKSGPITLLVQGHMEGEVWGLSTHPYLPICATVSDDKTLRIWDLSPSHC |
| 134 | 4.04E-06 | H2-IAd_Rad21 | Rad21 | Lys16Thr | LSKRGPLATIWLAAH | MFYAHFVLSKRGPLATIWLAAHWDKKLTKAHVFECNLESSVES |
| 135 | 3.53E-06 | H2-IAd_Rassf7 | Rassf7 | Ser90Arg | VQFVLRRTGPRLSGR | QECPVGAQATCGQFANDVQFVLRRTGPRLSGRPSSDNCPPPERCPVRASLPPKPS |
| 136 | 2.84E-06 | H2-IAd_Pnp_2 | Pnp | Asp248Glu | VFGFSLITNKVVMEY | TVPEVIVARHCGLRVFGFSLITNKVVMEYENLEKANHMEVLDAGKAAAQTLERFV |
| 137 | 2.33E-06 | H2-IAd_Rfc3_4 | Rfc3 | Arg213His | EGLALPSTLAHRLAE | APSIEDICSVLSTVCRKEGLALPSTLAHRLAEKSCRNLRKALLMCEACRVQQYPF |
| 138 | 2.27E-06 | H2-IAd_Ndufs6 | Ndufs6 | Val4Ala | AALTFRRLLTLPRAA | MAAALTFRRLLTLPRAARGFGVQVSPSGEKI |
| 139 | 2.05E-06 | H2-IAd_Polr3a | Polr3a | Val266Gly | LTRLLVPPLCIRPSG | LMNPESGKPSDLILTRLLVPPLCIRPSGVSDLKSGTNEDDLTMKLTEIIFLNDVI |
| 140 | 2.03E-06 | H2-IAd_Tctn1_4 | Tctn1 | Ala273Pro | PHYSSPAILRVPNSM | AFLVSQAFECSRRVDIEQCEGMEALSMPHYSSPAILRVPNSMTQVSIKIQSVMYR |
| 141 | 1.68E-06 | H2-IAd_Tmem39b | Tmem39b | Ala131Pro | IDFNLLMVTPIVLGR | WWYPPSHPPSHTSLNFHLIDFNLLMVTPIVLGRRFIGSIVKEASQRGKVSLFRSI |
| 142 | 7.63E-07 | H2-IAb_Tab2 | Tab2 | Ser151Thr | FNVFGMPSTSGASNT | QEPQTAPAQVPQGFNVFGMPSTSGASNTTPHLGFHLGSKGTSNLSQQTPRFNPIM |
| 143 | 5.98E-07 | H2-IAd_Wsb2_4 | Wsb2 | Pro390Gln | YQVLALQIPKKMKEF | PRVLSSLKHLCRKALRSFLTTYQVLALQIPKKMKEFLTYRTF |
| 144 | 3.41E-07 | H2-IAd_Prob1 | Prob1 | Pro811Ala | AAELETPLVAPATAV | SPLESTGTRPPRPGSPQACPNSSLRAAAELETPLVAPATAVQAPLLPDHPATAAR |
| 145 | 2.96E-07 | H2-IAd_Ftsj2 | Ftsj2 | Arg134Thr | TTFQKILELLPSRRA | FVLGVDLLHIFPLAGATFLCPADVTDPTTFQKILELLPSRRADVILSDMAPNATG |
| 146 | 2.94E-07 | H2-IAb_Dennd1a | Dennd1a | Ser799Pro | PGSTIPSHPATPSAA | PPSPAPGAAGTGSDALLALLDPLNTAWPGSTIPSHPATPSAATPFIPQLSFPPTV |
| 147 | 2.77E-07 | H2-IAd_Dhh_3 | Dhh | Asn157Asp | DKYGLLARLAVEAGF | DEDGHHAQDSLHYEGRALDITTSDRDRDKYGLLARLAVEAGFDWVYYESRNHIHV |

**Table S3. IEDB mapping of experimentally validated peptides.**

| Neo-intline^a^ | 45-mer Peptide | Core peptide | Evidence^b^ | COMPILED DATA |
| --- | --- | --- | --- | --- |
| MHC-I-1 | MGSLPEEKD**SALWSDTPM**GPLSAYRARASFNSGELLLFWD | SALWSDTPM | IEDB:1274321 | IFNg release |
| MHC-I-2 | RTGQNTWESKNILA**VSFAPLVQL**SKNDNGTPDSVGLPPCAVRNLQ | VSFAPLVQL | IEDB:1298990 | IFNg release |
| MHC-I-6 | PKYFKHNNMASFVRQLNM**YGFRNVVHI**ESGIIKQERDGPVEFQHP | YGFRNVVHI | IEDB:1304014 | IFNg release |
| MHC-I-11 | ESQVAGTPMFVVKAY**LPVNESFAF**TADLRSNTGGQAFPQCVFDHW | LPVNESFAF | Pubmed ID: 25901682 | Mouse vaccination *in vivo* |
| MHC-I-19 | YSITEGNIGEKFSMDHKTGT**IAMQNTTQL**RSRYELTVRASDGRFT | IAMQNTTQL | IEDB:1229791 | IFNg release/proliferation/qualitative binding |
| MHC-I-21 | NRIMSVVDPNHSGL**VTFQAFIDV**MSRETTDTDTADQVIASFKVLA | VTFQAFIDV | Pubmed ID: 25901682 | Mouse vaccination *in vivo* |
| MHC-II-6 | GNLFLPFLNVEIRDLRRP**GRYFLKSSSATETMH**FASRTRQSCISASASGLDTSSL | GRYFLKSSSATETMH | IEDB:1225117 | IFNg release |
| MHC-II-16 | IDYEKILKLTADAKFESGDVKATVA**VLGFILSSAAKHSVD**SDSLSSELQQLGLPK | VLGFILSSAAKHSVD | IEDB:1296009 | IFNg release |
| MHC-II-20 | ECGKHDRDLLIGTAKHGLNRT**DYYIMNGPQLSFLDA**YRNYAQHKRTDTQAPGSLC | DYYIMNGPQLSFLDA | IEDB:1201636 | IFNg release |

^a^Number of peptides screened by Neo-intline

^b^Evidence means the resource of previous reports that experimentally validated the identical peptides.

**Table S4. Overlapped peptides between model prediction and previous validation.**

| *Sequence* | *Evidence resources* | *Label* | *Patient_id^a^* |
| --- | --- | --- | --- |
| GADGVGKSAL | Pubmed ID: 26516200; IEDB ID: 1141716 | 1 | 4069;3995 |
| PYGYVLNEF | Pubmed ID: 29867227 | 0 | 4136 |
| MPYGYVLNEF | Pubmed ID: 29867227 | 1 | 4136 |
| YLSHLPLTCK | Pubmed ID: 26516200 | 0 | 4032 |
| RELVHRILL | Pubmed ID: 26516200 | 0 | 3995 |
| RVSTLRVSL | IEDB ID: 742513 | 1 | 3784 |
| HQNPVTGLLL | IEDB ID: 742503 | 1 | 3903 |
| KIQRNLWTL | IEDB ID: 157458 | 1 | 3903 |
| RARSRTPSC | Pubmed ID: 26901407 | 0 | 3784 |
| KVDPIGHVYI | Pubmed ID: 26901407 | 0 | 3998 |
| KVDPIGHVYIF | Pubmed ID: 26901407 | 1 | 3998 |
| APARLERRHSA | IEDB ID: 742497 | 1 | 3784 |
| GADGVGKSA | IEDB ID: 1141714 | 1 | 3995 |
| LMKVDPIGHVY | Pubmed ID: 26901407 | 1 | 3998 |
| SEHEGSGPEL | Pubmed ID: 26516200 | 1 | 4069 |
| KVDPIGHVY | Pubmed ID: 26901407; IEDB ID: 1079584 | 1 | 3998 |
| VSVQIISCQY | Pubmed ID: 26901407 | 1 | 3998 |
| TQQAVPLFSK | Pubmed ID: 26516200 | 0 | 4032 |
| LMMSLSALF | IEDB ID: 1074771 | 0 | 3903 |
| RHDLPPYRVYL | IEDB ID: 742510 | 1 | 3903 |
| RILLVAASY | Pubmed ID: 26516200 | 0 | 3995 |
| AYHSIEWAI | Pubmed ID: 26901407 | 1 | 3903 |
| KTYDTVHRHL | Pubmed ID: 29867227 | 1 | 4136 |
| TYDTVHRHL | Pubmed ID: 29867227 | 1 | 4136 |
| EHEGSGPEL | Pubmed ID: 26516200 | 1 | 4069 |
| VVGADGVGK | IEDB ID: 1310222 | 1 | 4069 |
| VVVGADGVGK | IEDB ID: 1309311 | 1 | 4069 |
| CVRVSGQGL | Pubmed ID: 26901407; IEDB ID: 1399225 | 1 | 3784 |
| NAYHSIEWAI | Pubmed ID: 26901407 | 1 | 3903 |
| IGDFGLATEK | IEDB ID: 1853234 | 0 | 3784 |
| LLQCTQQAV | Pubmed ID: 26516200 | 1 | 4032 |
| GLATEKSRW | IEDB ID: 2165196 | 1 | 3998 |
| ILCETCLIV | Pubmed ID: 26516200; IEDB ID: 1125072 | 1 | 4032 |
| RLLQCTQQAV | Pubmed ID: 26516200 | 1 | 4032 |
| GDFGLATEK | IEDB ID: 1853001 | 0 | 3784 |
| FVVPYMIYLL | IEDB ID: 1400125 | 1 | 3998 |
| RYTEGAELM | IEDB ID: 1821075 | 0 | 4032 |
| SQSPRSPPGK | IEDB ID: 1324923 | 0 | 4032 |
| YHSIEWAI | IEDB ID: 742520 | 1 | 3903 |
| VQIISCQY | IEDB ID: 1405954 | 1 | 3998 |
| FLGAGLFLYF | IEDB ID: 1087516 | 1 | 4032 |
| MDLLSSRFPL | IEDB ID: 2173873 | 1 | 3903 |
| KLVVVGADGV | IEDB ID: 1070772 | 1 | 4069 |
| KLVVVGAGGV | IEDB ID: 175610 | 0 | 4032 |
| YYPPSQIAQL | IEDB ID: 474330 | 1 | 4136 |
| LQCTQQAVPLF | Pubmed ID: 26516200 | 0 | 4032 |
| YLSHLPLTCKF | Pubmed ID: 26516200 | 0 | 4032 |
| SVQIISCQYL | Pubmed ID: 26901407 | 0 | 3998 |
| SMLRNERQFK | IEDB ID: 1825761 | 0 | 4032 |
| KSMLRNERQFK | IEDB ID: 1780915 | 0 | 4032 |
| VVVGAGGVGK | IEDB ID: 1301001 | 1 | 4032 |
| VVGAGGVGK | IEDB ID: 418684 | 1 | 4032 |
| RQVILCETCLI | Pubmed ID: 26516200 | 0 | 4032 |

^a^Patient ID is derived from the corresponding manuscript of the benchmark dataset.

**Supplementary Datas**

**Data S1.** Information of peptides validated in the TESLA community dataset.

**Data S2.** Indel annotation results of the whole exome sequencing. The dataset includes information of chromosomal localization, gene symbol, and nucleotide before and after the mutation, compared with the reference genome sequence.

**Data S3.** SNP annotation results of the whole exome sequencing. The dataset includes information of chromosomal localization, gene symbol, and nucleotide before and after the mutation, compared with the reference genome sequence.
